# A next generation setup for pre-fractionation of non-denatured proteins reveals diverse albumin proteoforms each carrying several post-translational modifications

**DOI:** 10.1038/s41598-019-48278-y

**Published:** 2019-08-13

**Authors:** Heidrun Rhode, Petra Muckova, Rita Büchler, Sindy Wendler, Bärbel Tautkus, Michaela Vogel, Thomas Moore, Julian Grosskreutz, Andree Klemm, Mary Nabity

**Affiliations:** 1 Institute of Biochemistry I, Nonnenplan 2-4, University Hospital Jena, 07740 Jena, Germany; 2 Analytik Jena, Konrad-Zuse-Str.1, 07745 Jena, Germany; 3 Department of Neurology, Am Klinikum 1, University Hospital Jena, 07747 Jena, Germany; 4 KfH Kuratorium für Dialyse und Nierentransplantation e.V., Ernst-Ruska-Ring 19, 07745 Jena, Germany; 5 Department of Veterinary Pathobiology, College of Veterinary Medicine, 4467 TAMU, Texas A&M University, College Station, TX 77843-4467 Texas, USA; 6 Present Address: Pharmachem Straße 1, Pharmachem Pößneck GmbH & Co. KG, 07381 Pößneck, Germany; 7 Present Address: Institute of Microbiology, Am Klinikum 1, University Hospital Jena, 07747 Jena, Germany

**Keywords:** Mass spectrometry, Liquid chromatography

## Abstract

Proteomic biomarker search requires the greatest analytical reproducibility and detailed information on altered proteoforms. Our protein pre-fractionation applies orthogonal native chromatography and conserves important features of protein variants such as native molecular weight, charge and major glycans. Moreover, we maximized reproducibility of sample pre-fractionation and preparation before mass spectrometry by parallelization and automation. In blood plasma and cerebrospinal fluid (CSF), most proteins, including candidate biomarkers, distribute into a multitude of chromatographic clusters. Plasma albumin, for example, divides into 15-17 clusters. As an example of our technique, we analyzed these albumin clusters from healthy volunteers and from dogs and identified cluster-typical modification patterns. Renal disease further modifies these patterns. In human CSF, we found only a subset of proteoforms with fewer modifications than in plasma. We infer from this example that our method can be used to identify and characterize distinct proteoforms and, optionally, enrich them, thereby yielding the characteristics of proteoform-selective biomarkers.

## Introduction

A major challenge in the search for proteomic biomarkers is the low reproducibility among studies. There are several likely reasons for this low reproducibility most of which originate from experimental design or methodology^1,2^ such as insufficient proteome coverage and low workflow precision^3,4^. It is also difficult to obtain longitudinal samples and perform multidisciplinary studies^1^. However, another significant cause is the biological variability of protein expression and modification^5–8^. Protein modifications are variable among healthy individuals and diseases further alter the diversity of multiple proteoforms^8^ producing dysfunctional or even toxic entities^9,10^. Therefore, it is illogical to expect that it is possible to produce a reliably usable biomarker from an entire protein family deduced from a few tryptic peptides^11,12^. This role is more reasonably fulfilled by one or multiple distinct proteoforms.

To identify and quantify specific proteoform biomarkers among the many and varied modifications possible, new experimental ideas and settings are required because the common shotgun and bottom-up approaches are insufficient for this purpose^13^. More suitable for identifying disease specific modifications are comprehensive, unbiased, and precise sample pre-fractionation methods on the protein level^7,8,12^, combined with sophisticated data analyses.

We therefore developed an unbiased protein pre-fractionation workflow using non-denaturing multidimensional chromatography. We adapted the workflow for the proteomic analysis of body fluids without any depletion. This workflow therefore allows the characterization of the entire proteome. The combination of parallel liquid handling, microplate format, and automation ensures maximum velocity, precision and reproducibility^14^. Moreover, the workflow inherently produces highly characteristic sub-fractions. This thereby preserves information on the native molecular weight (Mw, indicating complex formation, large posttranslational modifications (PTM), and fragmentation), charge (- PTM and conformation), and lectin affinity (- major types of glycans). The immune reactivity and biological activities of the proteoforms can also be analyzed in our native sub-fractions when required^15^. Such information is beneficial for identifying specific proteoform biomarkers as well as for their validation.

As expected, we showed that the majority of plasma proteins appear in multiple chromatographic clusters^7,16,17^. It is necessary to determine the probable reasons for this heterogeneity in order to identify biomarkers that are highly specific for particular diseases. A principal reason is that different proteoforms are characterized by different splice variants and PTM. In addition, however, it is necessary to consider the role of fragmentation and of complex formation with proteins and metabolites. Since albumin is the dominant plasma protein and is not known to be glycosylated or otherwise functionally modified but might be altered in the body by various processes^18^, we used it as an example for characterizing the capabilities of our workflow. To provide proof of principle, we searched albumin heterogeneity for various PTM.

## Results

### Characteristics of the separation procedure

**Figure 1:**
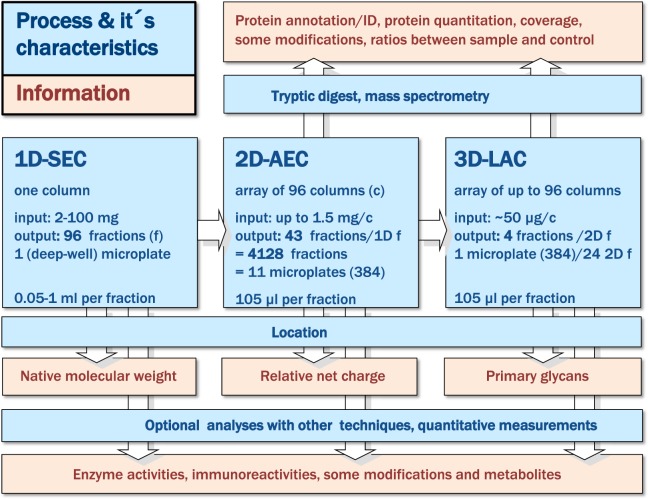
Scheme of the workflow. Blue: methods, processes and some characteristics; orange: potential information derived from the location of identified constituents and from additional analyses of sub-fractions.

**Figure 2:**
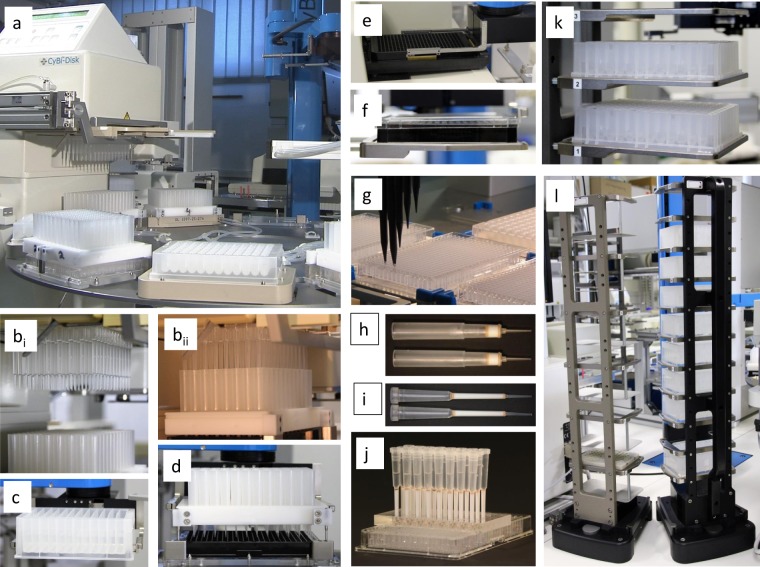
Devices and tools for automated and parallelized 2D-AEC and 3D-LAC. (**a**) Overview of our platform with the first liquid handler (used for the application of samples and elution solvents), the disc (carrying four 2D-column arrays and microplates containing 1D-fractions), and the robot for the automated handling of microplates, column arrays, tip racks, elution solvents, and microplate lids. (**b**–**l**) some details of chromatographic processing. (**b**) First liquid handler; 96 1D-fractions are pipetted onto the 2D (AEC)-column array (**b**_**i**_: tips above columns, **b**_**ii**_: tip position for discharging). (**c**) A reservoir containing one elution solvent is transported to the disc. (**d**) The column array is adjusted to the microplate. (**e**) Automatic read out of one 384-well microplate containing four 2D-fractions from each of the 96 1D-fractions; (**f**) Automatic sealing before moving into a refrigerator; (**g**) Second liquid handler; hit picking and reformatting of fractions; (**h**) Micro columns for 2D-AEC; (**i**) Micro columns for 3D-LAC; (**j**) Parallel 3D (LAC)-chromatography on 24 micro columns; (**k**) Stacker with two sample plates (96 1D-fractions of two samples); (**l**) Stacker for tip racks (left) and reservoirs of elution solvents (right).

Our method orthogonally combined size exclusion chromatography (SEC, first dimension, 1D), followed by anion exchange (AEC, 2D) and, optionally, lectin affinity (LAC, 3D) chromatography (Fig. 1). After serial 1D-fractionation, all further procedures were performed in microplate format. Beginning with 2D-fractionation, parallelization and automation was achieved for separation, preparation and analyses. Our automated workstation (Fig. 2a) enabled automated multichannel pipetting and robotic handling of microplates, reservoirs, and micro-column arrays, previously visualized by video^16^. For all fractionation processes, hit picking and analytics were controlled by adapted software packages. We developed several tools to maximize compatibility, throughput, recovery and precision of the preparation steps. We based protein recoveries from the corresponding load into liquid sub-fractions on UV measurements.

### Native size exclusion chromatography enabled high protein input

Our first step, 1D-SEC, was characterized by the greatest reproducibility for reference peaks of UV-absorbing material (Supplementary Fig. 1, Table 1) regarding height and retention (elution volume). This initial step determined the scale of protein input (from 2 to more than 100 mg) and the expected range of molecular weight (from 2 to 1000 kDa). As expected, the recovery of UV-absorbing material was ~100%. Thus, the entire protein content (>30 kDa) of blood plasma (mean 93.7, SD 2.7% of the UV-absorbing material, cf. Supplementary Fig. 1) was completely separated from the intrinsic low molecular weight peptidome (<6 kDa).

**Table 1:** Reproducibility of 1D-SEC.

Peak, valley	MW (kDa)	Major components	A_280_ (mAU)		elution volume (mL)	
			Mean	CV (%)	Mean	CV (%)
I	747.2	lipoproteins	558.8	0.93	49.01	0.00
1			168.0	1.99	54.73	0.12
II	203.2	immunoglobulins	1061.0	1.16	64.41	0.07
2			281.5	2.38	69.63	0.11
III	71.2	transferrin, albumin	1372.1	1.13	76.18	0.08
IV	2.3	intrinsic peptides	28.9	1.03	117.77	0.00
V	1.0	intrinsic peptides	275.92	5.47	127.13	0.06

Molecular weights were determined according column calibration^14^. For designation of peaks (maxima, I-V) and valleys (minima, 1, 2) see Supplementary Fig. 1. There were three measuring points around each maximum/minimum of each run (15 runs). The means and CV thus covered 45 data points (the same data as in Supplementary Fig. 1).

### Micro-column arrays provided truly parallel, automated and precise fractionation

No commercially available micro-columns for parallelized chromatography met all the requirements for volumes of 1D- and 2D-fractions, binding capacity, compatibility to 384-well microplates, free-flow elution of tunable elution rate, and precision. Therefore, we had to develop and optimize these tools ourselves. We made our own single columns and column arrays (Fig. 2d,j). We used empty SPE cartridges for AEC (Fig. 2h) and modified pipette tips for LAC (Fig. 2i). AEC runs over 100 µL Toyopearl DEAE-650M (Tosoh Bioscience GmbH, Stuttgart, Germany) and LAC over 70 µL Toyopearl AF-Tresyl 650 M (Tosoh Bioscience GmbH) modified with Concanavalin A (ConA, Sigma-Aldrich, C7275) and wheat germ agglutinin (WGA, Sigma-Aldrich, L9640)^14^. UV-absorbing material was recovered at mean 97.1, SD 11.2% with 3.8% CV after 2D fractionation and mean 88.8, SD 3.6% and 5.1% CV after 3D-fractionation^14^.

### Parallel medium exchange by dialysis reliably produced compatibility with subsequent steps

**Figure 3:**
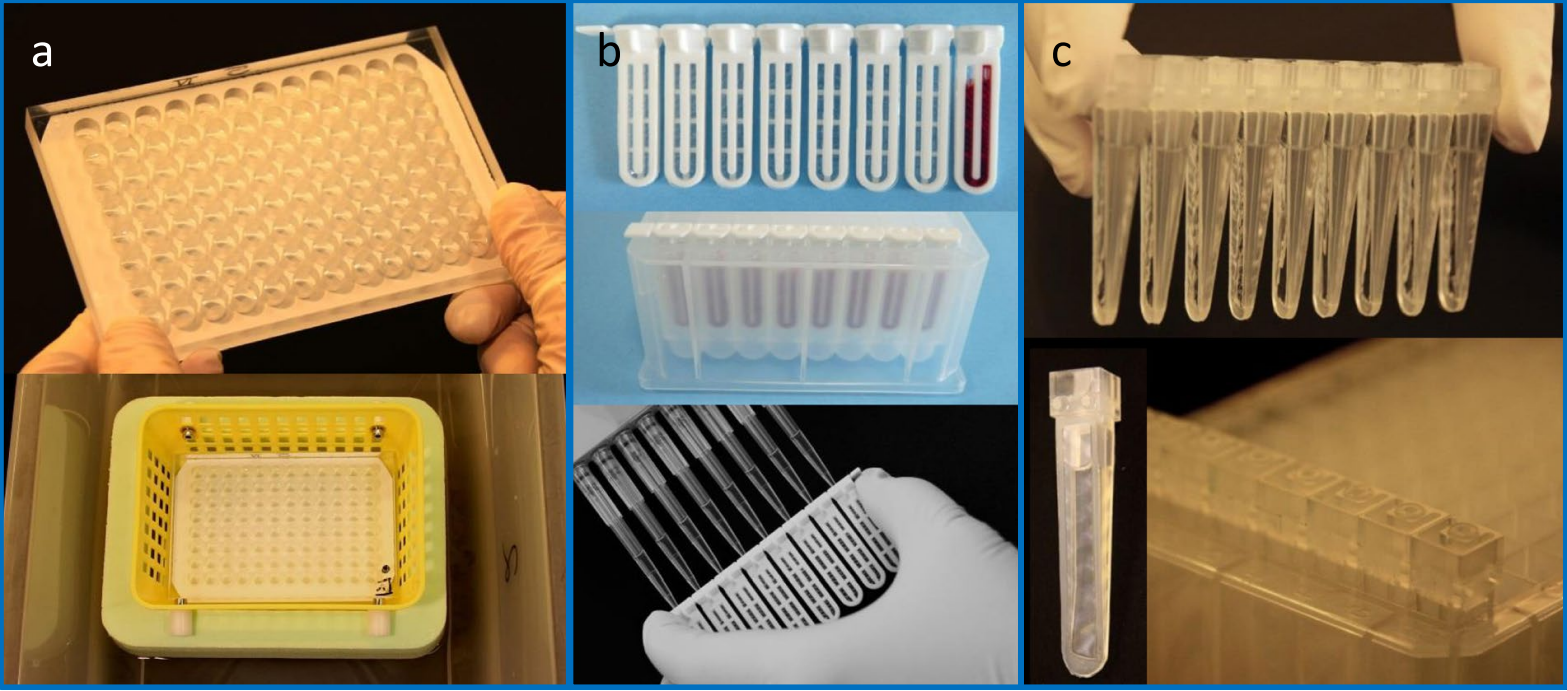
Parallel medium exchange applying three variants of suitable microdialysis tools. (**a**) Plate made in-house with 96 holes (250 µL) equipped with a semipermeable membrane (Merck Millipore, VCWP14250), applied for lowering the ionic strength of 1D-fractions before 2D-AEC; (**b**)Xpress Micro Dialyzer MD100 (Fa. Scienova Inc.^20,21^), ~30–100 µL, applied for the removal of reagents and medium exchange before tryptic digest and MS; (**c**) Microdialyzer (still prototypic^19^), ~10–550 µL, highest versatility, recovery and precision, applicable to all medium exchange steps of the entire workflow.

Dialysis ensured compatibility of 1D-sub-fractions with 2D-AEC. It also removed sugars from 3D-sub-fractions and superfluous reagents from all sub-fractions after denaturation and modification prior to tryptic digest and mass spectrometry (MS). To perform medium exchange, we selected parallelized microdialysis and developed various tools (Fig. 3)^19,20,14,21^ suitable for ensuring comprehensiveness and non-selectivity (see also https://www.scienova.com). For this method, only liquid handling was required, so guaranteeing high through-put, automation, and high precision. With the tool in Fig. 3a we routinely prepared 1D-sub-fractions for 2D-AEC. All tools achieved high protein recovery determined applying model proteins (bovine serum albumin (BSA), hemoglobin) and sample volumes of 100 µL recoveries (e.g. mean 93.1, SD 1.3%^21^ (tool in Fig. 3b); mean 100.8, SD 0.01%^19^ (tool in Fig. 3c)). Moreover, the tool in Fig. 3c was applicable to a wide range of sample volumes (from 10 to nearly 600 µL) with similar quality characteristics and it can be processed with various multichannel pipettes, including robots.

### Our automated platform executed laborious repeated cycles of parallel chromatography

**Figure 4:**
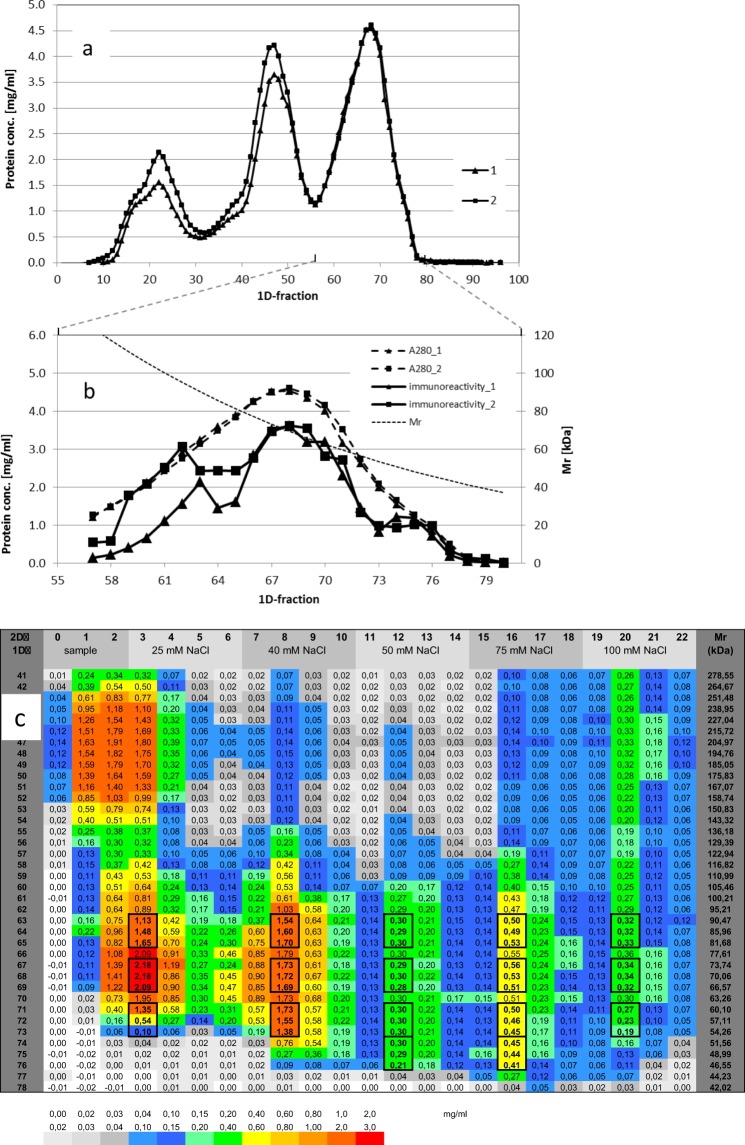
Protein separation after 1D-SEC and 2D-AEC. (**a**) Protein distribution after 1D-SEC of two samples from healthy male volunteers; section of all proteins above 30 kDa (A_280_, cf. Supplementary Fig. 1). One mL of EDTA plasma was injected and separated on a HiLoad Superdex™ 200 column (16/60, GE Healthcare) in 10 mM Tris-HCl containing 150 mM NaCl (pH 7.4) and 1 mM EDTA at 17 °C. After the void volume 96 fractions of 1 mL were collected using a flow rate of 1 mL/min. Column calibration was performed as previously described^14^. (**b**) Albumin concentrations after 1D-SEC determined by ELISA^15,26^; Section of 1D sub-fractions. (**c**) Section of 2D-sub-fractions containing serum albumin from sample 1 (see a). The entire protein distribution is reported elsewhere^14^. First column: number of 1D-fraction; last column: molecular weight according column calibration; first line: number of 2D-sub-fraction; second line: designation of sample and concentration of NaCl in the elution solvent 10 mM Tris-HCl, pH 7.4^14^. Frames indicate pooling from these fractions for PTM analysis. Total fractional concentrations of proteins were calculated from A_280_ using BSA as standard and are given by values (mg/mL) and color code.

We iteratively developed our automated platform^14,22^. With its current design (Fig. 2a) and its constituent tools (Figs 2 and 3), we were able to parallel process four samples through 2D-fractionation within three days (successive 1D SEC: two days including column calibration; parallel 2D AEC: one day including the medium exchange needed before AEC). Additional time was required for 3D-separation, tryptic digest, and MS. Our fractionation applied repeated process cycles with the selection of the target microplate, precise pipetting of fractions and of elution solvents on micro-columns, followed by complete flow-through and read out. 1D-sub-fractions were retrieved by the first liquid handler (Fig. 2a). The column arrays were adjusted to one partition of a microplate (Fig. 2d). The flow-through followed gravity without any support after a short initial pneumatic push (video in^16^). Elution solvents produced a stepwise gradient with increasing ionic strength^14^ (cf. Fig. 4c). From each 1D-fraction, four 2D-fractions were collected within one microplate. All successively filled microplates were instantly and automatically read (Fig. 2e), sealed, and transferred to the refrigerator (Fig. 2f). The platform can also, in principle, perform LAC. Picking and reformatting of fractions for subsequent preparation or MS was performed by the second liquid handler with 8 independent channels (Fig. 2g).

### Examples of verified biomarkers

Applications of our methods showed versatility and high protein yield of plasma proteins from humans, cattle, goats, dogs and mice, and from human CSF (cf. Supplementary Tab. 1). The number of proteins identified in human samples is similar to that of current studies applying various proteomic approaches (e.g.^23–25^). For example, from pooled human CSF, we were able to identify 5185 non-redundant proteins supported by ≥1 peptides and 1691 by ≥2 peptides. We also applied our method to the search for biomarkers of severe inflammation or sepsis, renal failure, Alport syndrome^17,26–28^, and psoriasis^16^. In these searches, we obtained several candidate markers with significantly altered protein concentrations inferred from MS data. We validated several of these using immunoassays.

It must be noted that we consistently identified not only one but several chromatographic clusters for the majority of altered proteins, some of which demonstrated opposite directions of alteration. Sometimes we were unable to verify the main alteration of such biomarker candidates by commercial ELISA^6,7^ rather than one out of a minor cluster^16^. We are currently searching for the probable reasons for this heterogeneity of our biomarkers. We were able, using the facilities of the Proteome Discoverer^©^ (see Online Methods), to uncover several interesting PTM for a variety of clusters of plasma proteins (patent sensitive data not shown). Below, we discuss our results for albumin, using it as an example of a typical eukaryotic protein.

### Albumin is a pure protein and not enzymatically modified but showing at least 17 proteoforms in blood plasma from human volunteers

We found albumin consistently highly heterogeneous in all of our blood samples from both patients and healthy control individuals^16,17,26,27^, just as many other plasma proteins are. After 1D-SEC, immune reactive albumin reproducibly separated into three distinct peaks with apparent molecular weights of ~90–95 kDa, ~69–73 kDa, and ~50 kDa (Fig. 4a,b). Moreover, the low Mw form showed the strongest decline in patients suffering from sepsis^26^. As many as 17 chromatographic clusters of this protein were produced after 2D-AEC (Fig. 4c). We analysed pools of 2D-sub-fractions of each cluster using the Proteome Discoverer^©^, as indicated by boxes (Fig. 4c) (cf. Methods).

### Each proteoform of serum albumin showed its own individual number and pattern of modifications

We were, in fact, able to identify several modifications, such as deamidation (R, N, Q), N-glycation (the majority on K, a few also predicted on R and Q), palmitoylation (S, K, T) and palmitoleylation (S, K), myristoylation (K), octanoylation (S, T), decanoylation (S, T), ethylation (E, K), acetylation (H, K, S, T, Y), methylation (D, E, H, I, K, L, N), dimethylation (K, N, R), propionylation (S, K), succinylations (K), carboxylation (K, E, D), carbamoylation (K), carboxymethylation (K), carboxyethylation (K), and phosphorylations (T, S, Y) and guanidylations (K).

**Figure 5:**
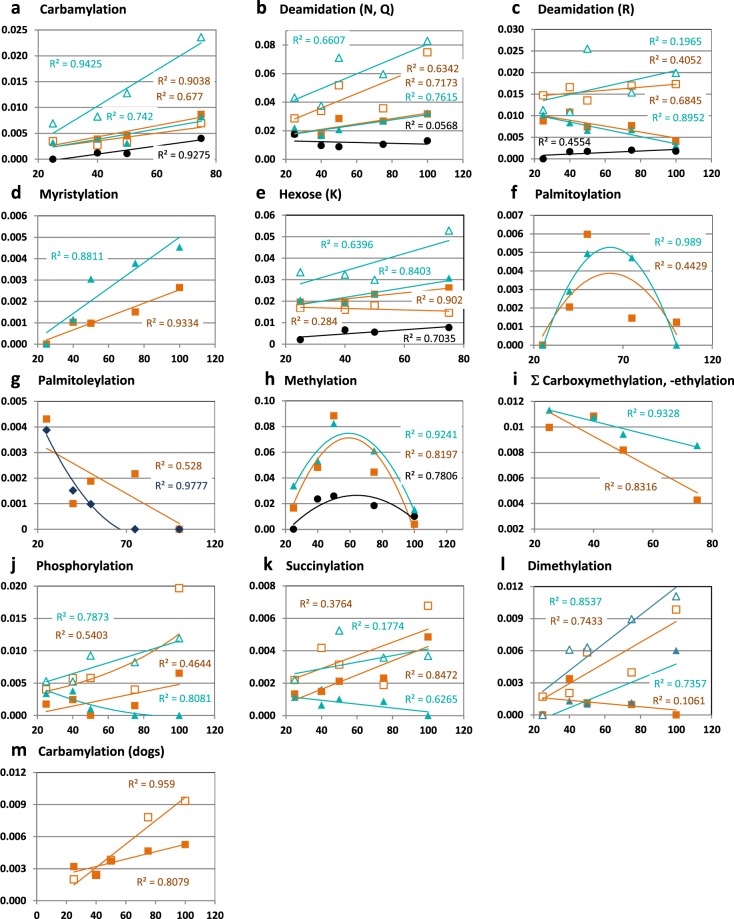
Dependence of the degree of PTM (n PTM/PSM) on the concentration of NaCl required for the elution of albumin from AEC matrix. Filled symbols: healthy plasma volunteers and healthy dogs; open symbols: plasma from patients with ESRD and dogs affected by preclinical Alport syndrome. Sub-fractions from plasma pools: orange squares, sub-fractions derived from albumin centric 1D (human plasma: 1D 67, dog plasma: 1D 23); turquoise triangles, sub-fractions derived from LMW albumin (1D 71); blue diamonds, sub-fractions derived from high Mw albumin (1D 63); black circles, sub-fractions from human CSF albumin, centric 1D 23. Data of human plasma were combined from individually pre-fractionated, prepared and analyzed samples from two healthy male volunteers (multi reports from four runs) and from four patients under hemodialysis (multi reports from eight runs). Due to slightly different pooling of the 2D-fractions, data from healthy male and female volunteers were not combined. Results from healthy female volunteers are similar to those from male volunteers and are shown in Supplementary Fig. 8. Accordingly, data from human CSF represent multi reports of nine runs (three sample pools analyzed in triplicate) and data from dog serum are means of technical duplicates from two sample pools. Ordinates: number of affected sites of the modification per PSM (n PTM/PSM) Abscissas: Concentration of NaCl (mM) required for elution of the sub-fraction from the AEC matrix (cf. Fig. 4c). R^2^ is given in the same color as and near to the corresponding data points.

To compare the results of different sub-fractions, the number of modified sites was normalized by the total number of identified peptide spectra matched for albumin (PSM) of the same search run. This allowed for a semi-quantitative comparison based on the degree of modification. As expected, the chromatographic clusters and the pools they represented were not uniformly modified. In healthy controls, each cluster possessed its own characteristic pattern of modification (Supplementary Fig. 2a). Moreover, in all blood samples, the degree of an assortment of modifications correlated well with the NaCl concentration required for AEC elution with respect to the predicted net charge, at least with the major albumin-containing pools that exhibited the highest PSM and sequence coverage. These modifications were carbamyolation (Fig. 5a), deamidation (N, Q: Fig. 5b; R: Fig. 5c), myristylation (Fig. 5d), and the number of hexoses linked to K (Fig. 5e). Carboxymethylation and carboxethylation inversely correlated with AEC elution (Fig. 5i). Palmitoylation, palmitoleylation, and methylation show maxima in different sub-fractions (Fig. 5f–h). Moreover, in healthy controls, the intensity of fluorescence related to the advanced glycation end products (AGE) correlated with the ionic strength (Supplementary Fig. 6a) required for elution as well as with carbamoylation and glycation (Supplementary Fig. 6b,c).

Albumin clusters in non-diabetic patients suffering from ESRD had a similar modification pattern (Supplementary Fig. 2b). Of note is the marked elevation of deamidations (Fig. 5b,c), phosphorylation, succinylation (Fig. 5j,k), as were carbamoylation. This was particularly so in sub-fractions originating from the apparently low Mw 1D-fraction (1D 71, Fig. 5a). As expected, AGE-fluorescence intensities were much higher in all sub-fractions from patients than from controls (Supplementary Fig. 6a). In contrast to the situation in healthy people (see above), in patients most of these values do not correlate either with the number of the same modifications per PSM or with carboxymethylation and methylation (Supplementary Fig. 6).

### Serum albumin of dog puppies is also heterogeneous in blood plasma

Plasma from puppies had a similar distribution of sub-fractions containing albumin (P49822, Supplementary Fig. 3). The centric Mw was ~6 kDa higher than predicted from the sequence data (Uniprot) as with human albumin. However, because of small volumes, it was not possible to build pools of the residual 2D-fractions. Therefore, we reanalyzed the raw data from single 2D-fractions derived from the centric 1D-fraction 64. These fractions contained several modifications similar to those in human albumin although all to a lower degree: carbamoylation (K) (Fig. 5l), deamidations (N, Q, R) N-glycation (K), methylation (E, D, H), and carboxymethylation (K) and acetylation (T, S, H, Y)). With the exception of carbamoylation, these modifications show no clear correlation with AEC-elution. In contrast to the case in samples from human adults, there was no palmitoylation, myristoylation, octanoylation or decanoylation. However, some propionylations on S471 were evident. It should be noted that the degree of carbamoylation of 2 sub-fractions was much higher in puppies with preclinical Alport syndrome (bearing the collagen IVα5 mutation) than in healthy controls (Fig. 5l).

### Albumin in human CSF

In contrast to plasma albumin, the main protein peak in CSF has only one centric Mw of ~78 kDa after 1D-SEC which, as in plasma, was distributed over five 2D-AEC elution steps (Supplementary Fig. 4). After immune quantitation, we were only able to identify a subset of only 4 albumin proteoforms in CSF (Supplementary Fig. 5). These had several modifications, such as carbamoylation (Fig. 5a), glycation (Fig. 5e), and methylation (Fig. 5h), which were correlated with the expected net charge. Nevertheless, these modifications were rarer than in the corresponding albumin forms from plasma. A few deamidations were present but without correlation with 2D-AEC elution (Fig. 5b,c). While one octanylation (S478) was always present, there was no carboxymethylation and no carboxyethylation, and only very rare palmitoylation and myristoylation.

## Discussion

The precision of sample pre-fractionation and proteome coverage of our workflow allows reliable sample comparison for biomarker search. We were therefore able to validate several biomarker candidates by ELISA^16,17,26,28^. Nevertheless, it was not possible to verify by immune assay some of our other biomarker candidates that appear in one of a multitude of chromatographic clusters^16,28^. We therefore analyzed the principle and capability of our pre-fractionation in order to determine whether such clusters represent enriched proteoforms varying in, and separated according to, characteristic PTM. To this end we analyzed, as an example, chromatographic clusters of albumin, after 2D-separation. We omitted 3D-separation and the analysis of glycans because albumin is not glycosylated^29^. Although we still used highly variable protein mixtures for PTM search, albumin represented the main component throughout. Within each search run it was possible to identify not only location but also the maximum number of occupied or modified sites. However, it was possible to estimate the rate of modification by normalizing these counts with PSM (i.e. the sum of modified and non-modified identified peptides).

The serum albumin of healthy individuals and patients contains several major modifications attributed to micro heterogeneity (oxidation, glycation and S-nitrosylation)^10,18,30^. Nevertheless, information on the extent and distribution of other modifications is rare. There are, though, two major and seven minor fractions identified and partially characterized by IEX/MS starting from Cohn fraction IV^31^. Our native 2D-fractionation method applied to whole blood plasma shows, however, that the complexity is, in fact, much higher. Serum albumin subdivides into at least 17 different parts all of which are modified in multiple ways. Most of the modifications, with the exception of deamidation, glycation and methylation, affect less than one percent of all the peptides identified (PSM). Nevertheless, their cumulative effect on charge and conformation might produce the characteristic sub-fractions observed. Carbamoylation (K), deamidation (N, Q), and glycation (K) lead to the loss of positive charges^32^, produce higher negative net charges, and correlate well with the ionic strength necessary for elution from our AEC matrix. In contrast, we found an inverse relationship for carboxymethylation and carboxyethylation (K), although these modifications introduce additional negative charges (Fig. 5i). Carboxylations (K, E, D) surprisingly show no relation to AEC elution. It is possible, therefore, that this is because these modifications either form intramolecular complexes, complexes with other plasma proteins, or complexes with divalent metal ions, thereby altering conformation and the interaction with the AEC matrix. Some known modifications of albumin have a strong influence on the structure and function of albumin^18,33,34^. All other PTM were present to an even lower degree on various sites and showed no clear relation to AEC-elution. For the first time we identified various acylations of distinct albumin sub-fractions. Although up to 6 fatty acids might bind non-covalently to albumin with high affinity^29,35^, no covalent lipidation has yet been described in the literature. Nevertheless, N- and O-fatty-acylations occur on intracellular proteins^36^. Moreover, there is some data on acylated functionally active proteins in blood plasma, such as growth hormone^37^ and ghrelin^38^. Acylation can be achieved by both, enzymatic and non-enzymatic^37,39^ and seems to be compatible with water solubility when fatty acids are buried within hydrophobic pockets^38^, like the non-covalently bound hydrophobic cargo of albumin. It is conceivable that acylation might promote protein-protein interaction. Moreover, we found no S-palmitoylation^40^ (C) probably due to the instability of this bond under DDT reduction before MS^37^. Albumin acylation still requires verification by other means.

In addition, we re-identified a low degree of carbamoylation in healthy individuals in accordance with a previous study^41^ as well as its increase (in a sub-set of our albumin fractions) under renal failure. This modification competes with glycation and is raised under diabetes, chronic kidney diseases, and inflammation^41,42^. Carbamoylation might occur artificially if urea is present in high concentrations (e.g. for denaturation)^43^. This is not true for our method and therefore such an artefact is highly improbable.

Deamidation is a common modification of several proteins occurring spontaneously, both *in vivo* and *in vitro*. Non-enzymatic deamidation of Q and N is considered to be both pathological and physiological, increasing protein negative charge, altering conformation, stability, longevity, and function^44^. Although Q is more resistant to deamidation^44^ than N (under physiological conditions its half-life is ten times that of N), we identified Q and N deamidations in similar proportions in our albumin fractions (e.g. mean number/PSM in all pools from healthy males: N, 0.0123; Q, 0.0117). Deamidation (N + Q) shows a strong correlation with AEC elution (Fig. 5b). We cannot exclude, however, that spontaneous N deamidation might be favored to a degree by the conditions of our tryptic digest after protein separation. High pH and temperature, particularly with the use of ammonium bicarbonate, is known to produce this modification^44^. However, because we uniformly applied these conditions to all sub-fractions, the differences identified cannot be explained as artifacts. The degree of R deamidation is higher in ESRD patients than in healthy individuals, but lower than those of Q and N (Fig. 5c). Deamidation converts R into either ornithine or citrulline^45^ thereby altering polarity but not charge. Although this modification can occur spontaneously under alkaline conditions^45^, the sites of deamidation were identical across all analyzed plasma sub-fractions: R264, R343, R542. The degree of oxidation of M correlates well with our AEC elution. Although the oxidation of albumin (C, M) is a common and wide spread reaction^46^ particularly as part of the antioxidant activity of albumin^47,48^ and under oxidative stress in renal diseases^49^, we omitted these data, because we are unable to exclude the possibility of some oxidation during sample preparation. In contrast, glycation is unlikely to result from *ex vivo* reactions^50^, since this non-enzymatic reaction exhibits low binding rate constants and requires high glucose concentration and temperature^51^. This reaction is unlikely in our study as all our samples were frozen quickly in liquid nitrogen. Moreover, after 1D-separation (SEC) the proteins were freed from non-bound sugars. Thus the degree of glycation appears to reflect natural modification.

All PTM were primarily identified as mass alteration of peptides by MS. Although distinct mass alterations might be attributed to different PTM, there is currently no alternative to MS for the majority of the PTM we identified. For partial verification we were able to quantify albumin concentration, glycation, and carbamoylation by the ELISA techniques available. The total albumin immune reactivity correlated well with the MS measure “area” for all the albumin peptides identified (Supplementary Fig. 7a) as well as with PSM in the concentration range of the majority of sub-fractions. As expected, PSM values were saturated at high protein concentrations (Supplementary Fig. 7b). However, quite surprisingly, neither the degree of glycation or of carbamoylation by MS was correlated with those determined by ELISA (Supplementary Fig. 7c,d). The percentages of glycation were similar with both methods; carbamoylation was much lower in ELISA than in MS. This illustrates a difficulty; the MS and ELISA results do not refer to exactly the same items. In contrast to ELISA, the MS determination of hexoses included not only glucose but also other sugars of the same Mw without identifying these other sugars. MS quantifies the number of modified residues, ELISA, however, detected the concentration of modified protein molecules. ELISA therefore probably either detected only some of the modifications or identified them independently of the variation in the number of modified sites per protein molecule. Nevertheless, the percentages of the immune reactive glycated albumin protein correlated well with AEC elution (Supplementary Fig. 7e) and AGE fluorescence intensities (Supplementary Fig. 7f). The percentages were higher in patients than in controls in accordance with^52,53^. Moreover, the total amount of albumin determined by ELISA in patients was shifted slightly from little modified to greatly modified entities compared to amounts in healthy people (Supplementary Fig. 5). We were unable to analyze pentosidin formation by our MS equipment, a well-known modification under ESRD^54^. So we tried to quantify this modification by ELISA in order to explain the high AGE fluorescence intensities in samples from patients. However, we were unfortunately unable to do this because our 2D fractions contained protein concentrations at the lowest and most unreliable levels that ELISA is able to detect.

The three apparently different molecular weights of plasma albumin could not be explained by PTM, since MS data does not provide strict stoichiometry. Nevertheless, besides PTM it is also possible that apparently large size proteoforms result from complex formation with members of the “albuminome”^55^. We unfortunately cannot discriminate complex formation from co-elution, since non-covalent, low affinity complexes might be dissolved after 2D-AEC. For example, we were, in fact, able to identify α-1 antitrypsin and vitamin D-binding protein in some sub-fractions co-eluting with albumin (Supplementary Fig. S2a,b) which are well-known to ligate with albumin^56,57^. In healthy individuals, we can exclude the possibility that huge fragmentation and truncation produced low-molecular weight forms because the sequence coverages of the apparently low and high Mw forms are practically identical. It is probable that different strengths of the interaction with the SEC matrix due to different sets of PTM^58^ might be attributed to different protein conformations producing low apparent Mws. We are not able to exclude nicked but adhering fragments. By such modification no altered Mw might be produced rather than an altered conformation. The allocation of albumin after 2D-AEC unexpectedly does not always follow the estimated net charge (cf. carboxylation and carboxymethylation). Moreover, the distribution into identical sub-fractions from patients and from healthy individuals occurs with different degrees of modification (e.g. deamidation, carbamoylation). Hence, the assignment of proteoforms might depend only in part on net charge but also on different conformations, probably due to a certain modification threshold, pattern or, even, an as yet unrecognized modification.

CSF albumin is heterogeneous and also, to a degree, physicochemically different from that in plasma^59^. Applying our workflow, we were only able to identify only 4 clusters with different degrees of PTM. The degree of carbamoylation (Fig. 5a), glycation (Fig. 5e) and methylation (Fig. 5h) is much lower than in plasma. The degree of acylation is so low that CSF albumin can be considered non-acylated. It is possible to explain these differences between CSF albumin and plasma albumin either by a selective transfer of only a subset of modified proteoforms from plasma into CSF^60^, by the absence of complex formation with the plasma “albuminome” or, probably, by synthesis within the central nervous system.

In contrast to Multiomics by SIMPLEX, the simultaneous profiling of proteins, lipids and other metabolites in initially denatured samples^61^, our method provides a native proteoform-resolved measurement addressing PTM and compound adducts. Thus, the separation principle of our workflow, combined with its quality characteristics, reproducibly produces highly distinctive protein fractions. As we demonstrated by example, our workflow is optimally suitable for the detection and analysis of differentially regulated proteoforms. Major proteins, like albumin, can be analyzed directly in sub-fractions from blood plasma as well as from CSF. Some of the albumin PTM we identified coincide with those found elsewhere (see above). Moreover, our precise micro-separation can be used for the native preparation of sufficient amounts of selected minor constituents directly from pre-analyzed sub-fractions. This serves as a prerequisite for their final enrichment and subsequent more reliable characterization by targeted MS or other analytical techniques so as to provide true biomarker candidates and antigens for the development of highly PTM selective immunological assays.

## Methods

### Plasma samples

We included 4 randomly selected healthy human volunteers (mean age 33.5, SD 5.9 years) and 4 patients (54.5 SD 2.3) with end stage renal failure but without diabetes mellitus. Both groups contained 2 males and 2 females. Nine milliliters of blood were collected by venipuncture into EDTA containers (02.1066.001, Sarstedt Nümbrecht, Germany) and centrifuged at 1500 g * 10 min at room temperature no longer than 30 min after withdrawal^16^. Samples from patients were identically obtained immediately before hemodialysis treatment. Dog samples were collected from a colony of dogs with X-linked hereditary nephropathy. These dogs have a naturally occurring, 10 base pair deletion in the gene encoding the α5 chain of type IV collagen. Samples were collected from 7-week old unaffected male and female, carrier female, and affected male siblings by jugular venipuncture. Blood was placed into a glass EDTA tube which was immediately placed on ice and centrifuged within 30 minutes of collection at 1500 g * 10 min at 4 °C. After centrifugation, the tubes were placed on ice, and plasma was removed, avoiding the buffy coat interface. The plasma was maintained on ice until frozen at −80 °C within 2 hours of initial blood collection. The samples were stored at −80 °C for two to three weeks before being shipped on dry ice and transferred to long-term liquid nitrogen storage. No freeze-thaw cycle was allowed. Human samples were collected in accordance with ethics agreement 4829–06/2016 (JUH Jena). The informed consent of all participants was obtained. Dog samples were collected in accordance with ethics agreements AUP IACUC 2013–0028 and 2016_0017 (TAMU Texas). The study protocol was reviewed and approved by the Texas A&M University Institutional Animal Care and Use Committee.

### CSF samples

We used CSF sample from 26 patients with neurological symptoms. CSF samples were collected in accordance to the ethical agreement 3633-11/2012 (JUH Jena). From all participants 12 mL of CSF were collected in polypropylene tubes (Greiner Bio-One 115261) following standard protocol (of the 12, the first 4 mL were used for laboratory measurements). The samples were kept on ice and immediately centrifuged at the bed side for 10 min. at room temperature (3500 g). Supernatants were aliquoted into cryovials (VWR, 479–1237), shock frozen in liquid nitrogen and stored at −150 °C. The CSF samples were characterized using key laboratory parameters. For MS analysis we used only samples without either blood contamination or blood-brain barrier disturbance. We assessed blood contamination by visual inspection for erythrocyte sediment and barrier disturbance by checking for erythrocytes, total protein, cell count and CSF:serum albumin ratio). To ensure adequate amounts of protein for analysis we created 3 mixtures. Each mixture was made up of equal volumes of CSF samples from 7–10 individuals. These pools were concentrated ~70–95-fold by ultrafiltration (AMICON^™^ Ultra, MWCO 3 kDa, Merck Millipore), followed by 2D fractionation as described below.

### Sample preparation

Pre-fractionation of blood samples was performed according to an existing detailed method for human plasma and serum^14,16,17^. Briefly, we used a 16/60 Highload Superdex^TM^ 200 column and an Äkta purifier (GE-Healthcare, Munich, Germany) and an automated workstation (laboratory setting, Analytik Jena AG, Fig. 2) for 1D-SEC and 2D-AEC. Initial 1D-SEC fractionation produced 96 1D-fractions of different native molecular weight from about 1 mL plasma. Subsequent 2D-AEC on arrays (Fig. 2d) of self-made microcolumns (Fig. 2h) separated each 1D-fraction into 43 2D-sub-fractions by applying a stepwise elution protocol^14^. Human plasma was separated individually. Clusters of albumin containing 2D-fractions of human plasma samples were pooled (Fig. 4c). To obtain sufficient material for the analysis of PTM in puppies we built two sample mixtures (0.75 mL) from unaffected female and affected male dogs. Each mixture contained equal volumes from three individual samples. For the determination of the total number of proteins, four mixtures were built from another set of unaffected male and female, carrier female, and affected male dogs (1.0 mL) from three individual samples each. Pre-fractionation of CSF samples was slightly modified. Briefly, 0.25 mL of mixed and concentrated CSF containing a protein amount of 8 mg (BCA Assay Kit, Pierce, 23227) were 1D-separated on a Superdex 200 GL column (10/300). Thus, 66 fractions of 350 µL each were collected into chilled 96-well deep well plates (flow rate 350 µL/min). Of these, 39 1D-fractions (instead of the 43 for plasma, protein concentration >0.05 mg/mL (A_280_)) were separated by 2D AEC.

#### Protein recovery from microdialyzers

BSA was quantified by A_280_ and hemoglobin by the cyanhemiglobin reference method.

#### AGE-fluorescence

Gemini XPS Microplate Reader, Molecular Device (ex 355/em 460 nm, Cutoff 455^34^) 100 µL sample volume, in microplates (655076, Greiner bio-one).

#### Mass spectrometry and PTM search

We performed tryptic digest and MS as previously described^14,16^. Briefly, the separation of tryptic peptides prior to mass spectrometry was performed on a Hypersil Gold UHPLC column (1.9 µm, 50 × 1.0 mm) using an Accela 1250 UHPLC system (both Thermo Fisher Scientific, USA) using binary gradient elution of the mobile phase. This gradient involved 0.1% formic acid in (A) water and in (B) acetonitrile at a flow rate of 150 µL min-1, throughout (0–1 min 5% B, 21 min 30% B, 24 min 40% B, 25 min 90% B, 25.1–26 min 90% B, 26.1–30 min 5% B). Tandem mass spectrometry (MS/MS) measurements were carried out on an LTQ Orbitrap Discovery (Thermo Fisher Scientific, USA) by positive heated electrospray ionization (H-ESI) at a vaporizer temperature of 200 °C. Sheath gas flow (30.0) and auxiliary gas flow (10.0) were used to dry the ionspray. The ionization voltage was set to 4.5 kV and the temperature of the ion transfer tube to 275 °C. The MS/MS system was operated in data-dependent TOP10 mode using 1 microscan. For this purpose, ions were monitored in the LTQ ion trap in full scan centroid mode at m/z 350–1700. The ten most intense ions were run through collision-induced dissociation for further orbitrap high resolution (30,000) analysis (profile data type). Wideband activation was used. The automatic gain control target value for the Orbitrap mass analyzer in full scan mode was 1.0 × 106. The LC–MS/MS was operated via the graphical interface of the Xcalibur software 2.1. Fractions from blood plasma and CSF were run in duplicate and triplicate followed by a blank (water) to avoid carryover. For quality control, a digested transferrin (human (Holo), SERVA Electrophoresis GmbH, 36756, 50 µg mL-1) was analysed four times per microplate.

We considered identical chromatographic 2D-sub-fractions (identical 1D- and 2D-location) to contain identical proteoforms of albumin due to their identical physicochemical properties. Because of this, we were able to combine, when equally pre-fractionated, counts of the PTM of proteins of identical 1D- and 2D-location but from different runs. We built up to 17 pools from 2D-subfractions of each plasma sample containing the major parts of visible albumin clusters. Due to slight differences in pooling of 2D-sub-fractions from healthy individuals, we processed the data sets from the 2 females separately from those of the two males. Although the samples were processed apart, we combined the data from all 4 patients under hemodialysis (2 females and 2 males) by taking the mean values. These values we then compared with the identically obtained data from the 2 healthy male individuals. CSF and dog plasma albumin were analyzed in MS data of 2D-fractions of known major albumin content without pooling thereby reanalyzing the MS raw data from human CSF and dogs (still unpublished data) from other studies. The search for PTM ran successively using Proteome Discoverer^©^ 1.3 (Thermo) with no more than three dynamic modification types per run. We considered only highly confident database matches. The following dynamic modifications of amino acids were analyzed: oxidation (M), palmitoyl (K, S, T), palmitoleyl (S, T), myristoyl (K), octanoyl (S, T), decanoyl (S, T), deamidation (N, Q, R), carboxy (D, E, K, W), carboxymethyl (K, W), carboxyethyl (K), carbamoyl (K, M, R), hexose (K, N, R, T, W, Y), acetyl (H, K, S, T, Y), ethyl (E, K), dimethyl (K, N, R), propionyl (K, S), methyl (D, E, H, I, K, L, N), phosphoryl (S, T, Y), and succinyl (K). Multi reports of the related replicates were utilized.

#### ELISA

Albumin was measured using a slight modification of an existing method^26^. Briefly, 25 µL of the samples and the standards were incubated for 90 min. at room temperature in the wells of 384-well microplates (781094, Greiner bio-one) pre-coated with anti-human albumin (1.0 µg/mL (rabbit, DAKO) overnight at 4 °C). After washing (seven times, 100 µL) the wells were incubated with 25 µL goat anti-human albumin-HRP conjugate (1:20.000, A80-2299, Bethyl Lab.) for 2 h. After washing, 25 µL of substrate were added (TMB X-tra, Biotrend: #4800 H) and the reaction was stopped by 25 µL 0.5 M H_2_SO_4_ after ~20 min and visual inspection. Due to the limited sample volumes and protein concentrations, it was only possible to analyze the 8 most highly concentrated 2D-pools (67.1 to 67.4, 71.1 to 71.4) by **P**TM-selective ELISA according to the instruction of the suppliers. We tested all selected 2D-pools in duplicate with deviations of no more than 10%. Carbamoylation: Human Carbamylated Albumin ELISA (XPEH0930) supplied by XpressBio; 1 to 5 and 1 to 10 diluted 2D-pools. Pentosidine: Human Pentosidine ELISA-Kit (CSB-E09415h) from Cusabio® applying 1 to 5 diluted 2D-pools. Glycation: Glycated human albumin competitive ELISA-Kit (CSB-E09599h) from Cusabio®; slightly modified: we pre-mixed 2D-pools (diluted 1 to 3) and conjugate solutions before spotting to the wells.

## Supplementary information

Supplementary Figures and Table
