# Supplementary material for: A next generation setup for pre-fractionation of non-denatured proteins reveals diverse albumin proteoforms each carrying several post-translational modifications
Source: Sci Rep. 2019 Aug 13;9:11733. doi: 10.1038/s41598-019-48278-y (PMC6692309; doi:10.1038/s41598-019-48278-y)
Supplement: Supplementary file 1 — Supplementary Figures and Table [file 41598_2019_48278_MOESM1_ESM.pdf]

Supplementary Material

A next generation setup for pre-fractionation of non-denatured proteins reveals diverse albumin proteoforms each carrying several post-translational modifications

Heidrun Rhode, Petra Muckova, Rita Büchler, Sindy Wendler, Bärbel Tautkus, Michaela Vogel, Thomas Moore, Julian Grosskreutz, Andree Klemm, Mary Nabity

Fig. 1: Reproducibility of 1D-SEC of human plasma – overlay of 15 runs of an identical sample

Chromatography conditions and calibration: see Methods, Fig. 4a of the manuscript and [14].
Absorbance track at 280 nm; peaks and valleys are indicated by Roman and Arabic numbers and are used in Table 1 (main text).
Major components: in I, lipoproteins; in II, immunoglobulins; in III, albumin; in IV and V, intrinsic peptides of ~2-3 and ~1 kDa.

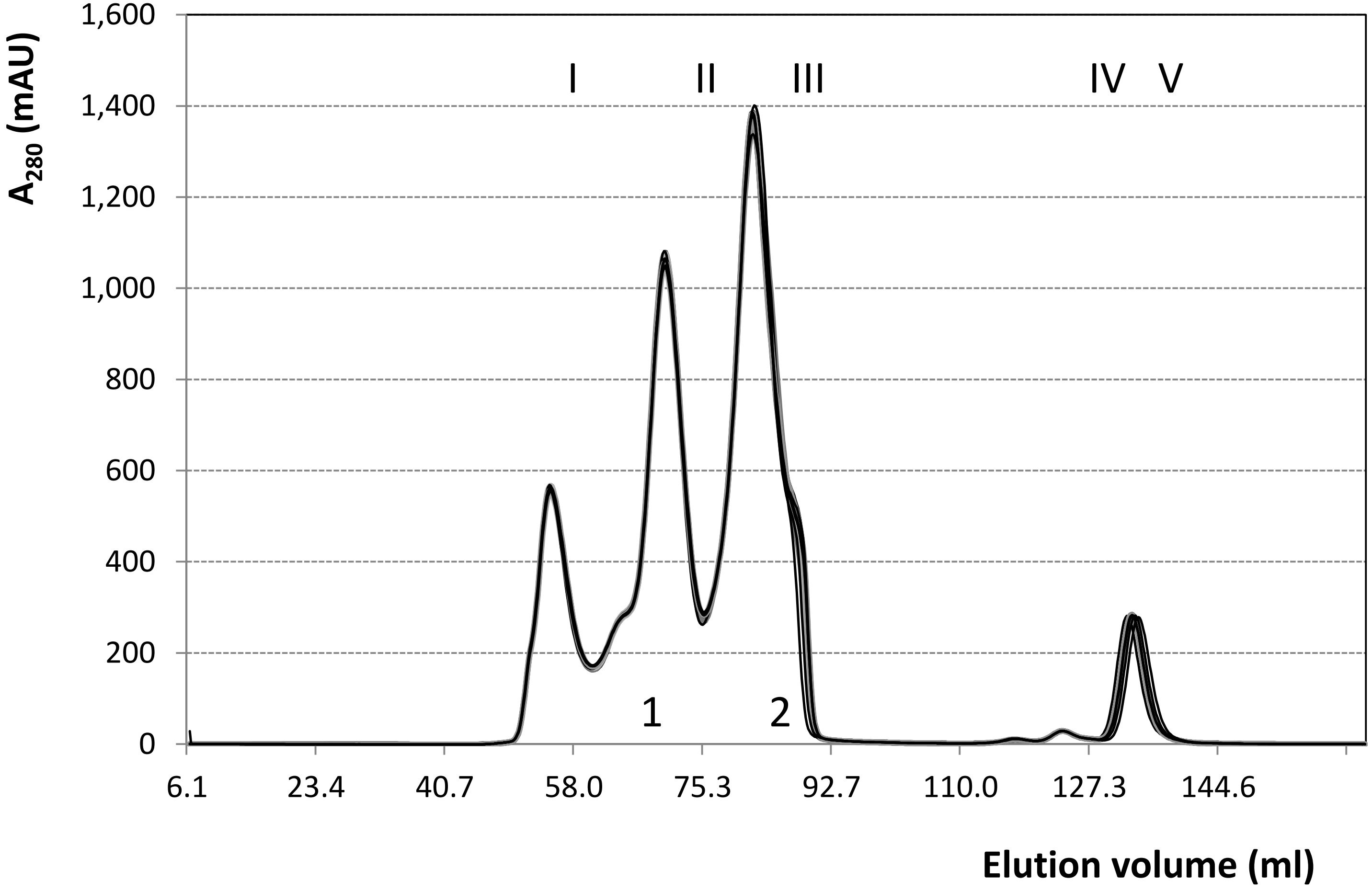

Table 1: Number of non-redundant proteins identified in 2D-sub-fractions applying our workflow to various sample types and amounts

Number of proteins supported by		Species	Sample type	Number of mixtures/individuals	Starting Protein amount (mg)
≥ 1 peptide	≥ 2 peptides				
4997	816	man	plasma	-/12	60-90
5185	1691	man	CSF	6/51	8
4194	592	cattle*	plasma	-/3	60-80
1044	218	goat*	plasma	-/1	75
3116	443	dog**	plasma	4/12	65-67
2542	434	mouse	serum	6/24	25-29

With man and mouse more than 50,000 data base entries were available at the time of analyses.
*: only 5000-7150 (2014-17) and ** ~2280 (2018) reviewed data base entries at the time of analyses (2015-2/2018).
All blood samples have been mass spec analyzed as technical duplicates, CSF samples as triplicates (cf. Methods).

Fig. 2a: PTM of albumin (P02768) in the pools of 2D-sub-fractions from human plasma from two male healthy volunteers. The numbers of 1D and 2D sub-fractions (cf. Fig. 4c) are given in the left column and upper line. Pools were built from three sub-fractions each, denominated in the first section of this Figure and and used also in Supplementary Fig. 5. Only three dynamic PTM were determined simultaneously by the Proteome Discoverer© multiconsensus analysis of four runs (two samples, analyzed in duplicates each, cf. Methods). The type of PTM is given in the head of each section. Data given are the numbers (#) of **modified residues per PSM** (total number of identified peptide spectra matched for albumin). The data are further visualized by a grey code.

Nomenclature of pools of three 2D sub-fractions each

2D→	3	8	12	16	20
1D↓					
62					
63					
64	63.1	63.2	63.3	63.4	63.5
65					
66					
67					
68	67.1	67.2	67.3	67.4	67.5
69					
70					
71					
72	71.10	71.2	71.3	71.4	71.5
73					
74					
75			75.1	75.2	
76					
77					

# Palmitoyl

2D→	3	8	12	16	20
1D↓					
62					
63					
64	0.0000	0.0065	0.0041	0.0016	0.0031
65					
66					
67					
68	0.0031	0.0021	0.0060	0.0015	0.0012
69					
70					
71					
72	0.0013	0.0029	0.0049	0.0047	0.0000
73					
74					
75			0.0032	0.0050	
76					
77					

Sum [# methylation, # dimethylation, # ethylation, # propionylation, and # acetylation]

2D→	3	8	12	16	20
1D↓					
62					
63					
64	0.099	0.063	0.038	0.095	0.029
65					
66					
67					
68	0.023	0.058	0.104	0.056	0.012
69					
70					
71					
72	0.039	0.063	0.094	0.072	0.033
73					
74					
75			0.063	0.039	
76					
77					

Sum [# octanoyl, # decanoyl, # myristoyl, # palmitoyl, # palmitoleyl]

2D→	3	8	12	16	20
1D↓					
62					
63					
64	0.0057	0.0150	0.0082	0.0031	0.0094
65					
66					
67					
68	0.0093	0.0056	0.0098	0.0058	0.0051
69					
70					
71					
72	0.0038	0.0068	0.0080	0.0130	0.0057
73					
74					
75			0.0076	0.0079	
76					
77					

# Myristoyl

2D→	3	8	12	16	20
1D↓					
62					
63					
64	0.0000	0.0046	0.0032	0.0008	0.0049
65					
66					
67					
68	0.0000	0.0010	0.0010	0.0015	0.0026
69					
70					
71					
72	0.0000	0.0012	0.0030	0.0038	0.0045
73					
74					
75			0.0043	0.0030	
76					
77					

Sum # deamidations of N and Q

2D→	3	8	12	16	20
1D↓					
62					
63					
64	0.0221	0.0205	0.0298	0.0225	0.0250
65					
66					
67					
68	0.0177	0.0200	0.0311	0.0318	0.0346
69					
70					
71					
72	0.0222	0.0192	0.0225	0.0260	0.0313
73					
74					
75			0.0238	0.0156	
76					
77					

Deamidation of R

2D→	3	8	12	16	20
1D↓					
62					
63					
64	0.0071	0.0114	0.0096	0.0092	0.0062
65					
66					
67					
68	0.0070	0.0106	0.0088	0.0078	0.0080
69					
70					
71					
72	0.0110	0.0088	0.0065	0.0052	0.0045
73					
74					
75			0.0085	0.0046	
76					
77					

Hexose on K

2D→	3	8	12	16	20
1D↓					
62					
63					
64	0.021	0.021	0.023	0.027	0.028
65					
66					
67					
68	0.020	0.020	0.023	0.026	0.025
69					
70					
71					
72	0.021	0.019	0.023	0.031	0.021
73					
74					
75			0.023	0.031	
76					
77					

Sum [# carboxymethyl and # carboxyethyl on K]

2D→	3	8	12	16	20
1D↓					
62					
63					
64	0.0118	0.0079	0.0114	0.0058	0.0073
65					
66					
67					
68	0.0100	0.0109	0.0082	0.0043	0.0058
69					
70					
71					
72	0.0113	0.0108	0.0094	0.0086	0.0044
73					
74					
75			0.0069	0.0085	
76					
77					

Carbamoyl on K

2D→	3	8	12	16	20
1D↓					
62					
63					
64	0.0017	0.0020	0.0010	0.0067	0.0056
65					
66					
67					
68	0.0034	0.0039	0.0046	0.0087	0.0058
69					
70					
71					
72	0.0032	0.0039	0.0031	0.0082	0.0035
73					
74					
75			0.0030	0.0046	
76					
77					

Fluorescence intensity at 460 nm (ex 355 nm)/(mg/ml) protein conc.

2D→	3	8	12	16	20
1D↓					
62					
63					
64	16.04	22.61	39.63	42.17	40.59
65					
66					
67					
68	16.64	22.42	52.62	59.49	53.79
69					
70					
71					
72	36.30	37.01	49.52	65.13	69.30
73					
74					
75			49.83	58.64	
76					
77					

Concentration parameters derived from A_{280}, ELISA and MS counts

Ratio (%) of concentrations of albumin (ELISA)/total protein (A_{280})

2D→	3	8	12	16	20
1D↓					
62					
63					
64	49.30	100.14	72.85	88.57	36.05
65					
66					
67					
68	99.36	105.90	57.70	60.13	23.20
69					
70					
71					
72	87.09	93.28	61.90	75.42	26.07
73					
74					
75			69.27	74.05	
76					
77					

Total protein concentration (mg/ml, A_{280})

2D→	3	8	12	16	20
1D↓					
62					
63					
64	1.54	1.67	0.38	0.61	0.34
65					
66					
67					
68	3.83	1.99	0.41	0.69	0.45
69					
70					
71					
72	1.22	1.81	0.40	0.56	0.32
73					
74					
75			0.38	0.47	
76					
77					

Sequence coverage of albumin, mean and (SD) (%)*

2D→	3	8	12	16	20
1D↓					
62					
63					
64	83.19 (1.09)	86.11 (0.96)	78.66 (1.39)	80.32 (1.13)	73.66 (1.14)(
65					
66					
67					
68	85.63 (1.50)	84.43 (1.55)	80.26 (1.61)	79.44 (1.63)	76.69 (1.38)
69					
70					
71					
72	84.30 (0.80)	81.63 (1.06)	78.79 (1.14)	77.55 (1.39)	73.50 (1.46)
73					
74					
75			74.19 (1.75)	74.36 (2.11)	
76					
77					

PSM (albumin), mean and (SD)*

2D→	3	8	12	16	20
1D↓					
62					
63					
64	723.6 (37.5)	1014.4 (76.4)	491.9 (36.7)	649.2 (28.3)	326.1 (16.7)
65					
66					
67					
68	1138.9 (59.1)	998.5 (45.9)	512.4 (38.4)	669.2 (30.0)	369.2 (22.6)
69					
70					
71					
72	819.2 (38.8)	855.5 (47.4)	507.1 (33.8)	554.5 (30.0)	335.9 (23.1)
73					
74					
75			476.6 (39.3)	545.4 (33.6)	
76					
77					

Protein of secondly highest abundance within the same pool (according coverage and area)

2D→	3	8	12	16	20
1D↓					
62					
63					
64	Trf	VDBP	A1AT ApoAI	A1AT	ApoAI A1AT
65					
66					
67					
68	Trf	VDBP	A1AT	A1AT	A1AT
69					
70					
71					
72	Trf VDBP	VDBP	VDBP	A1AT ApoAI	A1AT
73					
74					
75			VDBP	ApoAIV	
76					
77					

Abbreviations:

Trf, transferrin; Apo, apolipoprotein; A1AT, alpha-1-antitrypsin; VDBP, vitamin D-binding protein;

*Mean and standard deviation of coverages and PSMs after 9 search runs included here.

Fig. 2b: PTM of albumin (P02768) in the pools of 2D-sub-fractions from human plasma from non-diabetic ESRD patients undergoing hemodialysis. The numbers of 1D and 2D sub-fractions (cf. Fig. 4c) are given in the left column and upper line. Pools were built from three sub-fractions each, denominated in the first section of this Figure and are used also in Supplementary Fig. 5. Only three dynamic PTM were determined simultaneously by the Proteome Discoverer© by multiconsensus analysis of eight runs (four samples, analyzed in duplicates each, cf. Methods). The type of PTM is given in the head of each section. Data given are the numbers (#) of **modified residues per PSM** (total number of identified peptide spectra matched for albumin). The data are further visualized by a grey code.

Nomenclature of pools of three 2D sub-fractions each

2D→	3	8	12	16	20
1D↓					
62					
63					
64	63.1	63.2	63.3	63.4	63.5
65					
66					
67					
68	67.1	67.2	67.3	67.4	67.5
69					
70					
71					
72	71.10	71.2	71.3	71.4	71.5
73					
74					
75			75.1	75.2	
76					
77					

Sum [#octanoyl, # decanoyl, #myristoyl, #palmitoyl, #palmitoleyl]

2D→	3	8	12	16	20
1D↓					
62					
63					
64	0.0024	0.0051	0.0035	0.0004	
65					
66					
67					
68	0.0068	0.0036	0.0066	0.0033	0.0038
69					
70					
71					
72	0.0057	0.0097	0.0062	0.0077	0.0214
73					
74					
75			0.0095	0.0043	
76					
77					

# Palmitoyl

2D→	3	8	12	16	20
1D↓					
62					
63					
64	0.0000	0.0018	0.0005	0.0000	
65					
66					
67					
68	0.0034	0.0000	0.0018	0.0004	0.0013
69					
70					
71					
72	0.0024	0.0048	0.0041	0.0016	0.0078
73					
74					
75			0.0022	0.0000	
76					
77					

# Myristoyl

2D→	3	8	12	16	20
1D↓					
62					
63					
64	0.000	0.001	0.000	0.000	
65					
66					
67					
68	0.001	0.000	0.002	0.001	0.003
69					
70					
71					
72	0.001	0.002	0.001	0.002	0.012
73					
74					
75			0.001	0.002	
76					
77					

Sum [#methylation, #dimethylation, #ethylation, #propionylation, and #acetylation]

2D→	3	8	12	16	20
1D↓					
62					
63					
64	0.0238	0.0255	0.0411	0.0372	
65					
66					
67					
68	0.0224	0.0201	0.0285	0.0271	0.0480
69					
70					
71					
72	0.0519	0.0559	0.0499	0.1005	0.0508
73					
74					
75			0.0659	0.1104	
76					
77					

Sum #deamidations of N and Q

2D→	3	8	12	16	20
1D↓					
62					
63					
64	0.038	0.032	0.048	0.034	
65					
66					
67					
68	0.029	0.034	0.052	0.036	0.075
69					
70					
71					
72	0.043	0.037	0.071	0.060	0.083
73					
74					
75			0.077	0.065	
76					
77					

# Deamidation of R

2D→	3	8	12	16	20
1D↓					
62					
63					
64	0.010	0.017	0.016	0.016	
65					
66					
67					
68	0.015	0.017	0.014	0.017	0.017
69					
70					
71					
72	0.011	0.011	0.026	0.015	0.020
73					
74					
75			0.017	0.023	
76					
77					

# Hexose on K

2D→	3	8	12	16	20
1D↓					
62					
63					
64	0.013	0.016	0.024	0.026	
65					
66					
67					
68	0.017	0.016	0.018	0.015	0.035
69					
70					
71					
72	0.033	0.032	0.030	0.053	0.025
73					
74					
75			0.032	0.032	
76					
77					

# Succinyl

2D→	3	8	12	16	20
1D↓					
62					
63					
64	0.0028	0.0018	0.0053	0.0022	
65					
66					
67					
68	0.0022	0.0042	0.0031	0.0019	0.0068
69					
70					
71					
72	0.0022	0.0016	0.0052	0.0036	0.0037
73					
74					
75			0.0036	0.0023	
76					
77					

# Carbamoyl on K

2D→	3	8	12	16	20
1D↓					
62					
63					
64	0.0028	0.0037	0.0016	0.0099	
65					
66					
67					
68	0.0035	0.0027	0.0032	0.0070	0.0145
69					
70					
71					
72	0.0069	0.0082	0.0128	0.0236	0.0116
73					
74					
75			0.0064	0.0092	
76					
77					

# Phosphorylation

2D→	3	8	12	16	20
1D↓					
62					
63					
64	0.0074	0.0004	0.0017	0.0037	
65					
66					
67					
68	0.0040	0.0058	0.0058	0.0040	0.0197
69					
70					
71					
72	0.0053	0.0052	0.0092	0.0082	0.0119
73					
74					
75			0.0011	0.0091	
76					
77					

Sum [# carboxymethyl and # carboxyethyl on K]

2D→	3	8	12	16	20
1D↓					
62					
63					
64	0.0075	0.0120	0.0119	0.0078	
65					
66					
67					
68	0.0078	0.0111	0.0181	0.0069	0.0207
69					
70					
71					
72	0.0079	0.0116	0.0141	0.0115	0.0112
73					
74					
75			0.0167	0.0153	
76					
77					

Fluorescence intensity at 460 nm (ex 355 nm)/(mg/ml) protein conc.

2D→	3	8	12	16	20
1D↓					
62					
63					
64	46.3	114.6	153.1	212.6	
65					
66					
67					
68	109.2	147.7	179.1	234.7	167.1
69					
70					
71					
72	74.6	104.0	158.5	216.4	148.5
73					
74					
75			102.4	169.0	
76					
77					

Concentration parameters derived from A_{280}, ELISA and MS counts

Ratio (%) of concentrations of albumin (ELISA)/total protein (A_{280})

2D→	3	8	12	16	20
1D↓					
62					
63					
64	36.56	103.55	87.24	81.36	
65					
66					
67					
68	108.12	102.14	90.64	51.20	25.31
69					
70					
71					
72	72.84	82.74	89.43	76.46	28.24
73					
74					
75			84.66	51.87	
76					
77					

Total protein concentration (mg/ml, A_{280})

2D→	3	8	12	16	20
1D↓					
62					
63					
64	1.260	1.314	0.329	0.513	
65					
66					
67					
68	2.125	1.595	0.358	0.623	0.375
69					
70					
71					
72	1.175	1.559	0.358	0.539	0.312
73					
74					
75			0.339	0.407	
76					
77					

Sequence coverage of albumin, mean and (SD) (%)*

2D→	3	8	12	16	20
1D↓					
62					
63					
64	91.66 (0.63)	94.18 (0.52)	91.85 (0.99)	92.47 (0.68)	
65					
66					
67					
68	93.50 (0.47)	93.32 (0.45)	90.03 (0.65)	93.29 (0.53)	68.33 (3.41)
69					
70					
71					
72	78.39 (1.62)	79.08 (1.88)	71.23 (2.10)	72.93 (1.91)	61.30 (2.98)
73					
74					
75			63.58 (1.99)	68.05 (2.07)	
76					
77					

PSM (albumin), mean and (SD)*

2D→	3	8	12	16	20
1D↓					
62					
63					
64	533.2 (54.15)	742.9 (88.20)	449.6 (49.87)	646.8 (54.30)	
65					
66					
67					
68	759.6 (75.64)	655.0 (68.02)	362.7 (36.59)	598.5 (55.27)	193.8 (27.28)
69					
70					
71					
72	465.4 (40.03)	523.1 (48.10)	240.3 (24.84)	287.8 (30.18)	152.3 (21.28)
73					
74					
75			217.1 (23.63)	234.5 (25.18)	
76					
77					

Protein of secondly highest abundance within the same pool (according coverage and area)

2D→	3	8	12	16	20
1D↓					
62					
63					
64	Trf	Trf ApoAI	ApoAI	A1AT	
65					
66					
67					
68	Trf	Trf	Trf	Trf	A1AT A1AGP
69					
70					
71					
72	Trf VDBP	VDBP	ApoAIV	ApoAIV	A1AT
73					
74					
75			ApoAIV	ApoAIV	
76					
77					

Abbreviations:

Trf, transferrin; Apo, apolipoprotein; A1AT, alpha-1-antitrypsin; A1AGP, alpha-1-acid glycoprotein; VDBP, vitamin D-binding protein;

*Mean and standard deviation of coverages and PSMs after 9 search runs included here.

Fig. 3: Protein distribution of dog plasma after 2D-AEC, section of albumin containing sub-fractions

2D→ 1D↓	0	1	2	3	4	5	6	7	8	9	10	11	12	13	14	15	16	17	18	19	20	21	22	23	24	25	26	Mr (kDa)
	sample				25 mM NaCl				40 mM NaCl				50 mM NaCl				75 mM NaCl				100 mM NaCl				125 mM NaCl			
52	0,01	0,03	0,13	0,19	0,02	0,01	0,01	0,01	0,05	0,04	0,02	0,02	0,04	0,03	0,02	0,02	0,08	0,03	0,02	0,03	0,25	0,20	0,11	0,07	0,12	0,03	0,02	146,95
53	0,00	0,04	0,15	0,21	0,02	0,01	0,01	0,01	0,05	0,04	0,02	0,02	0,04	0,03	0,02	0,02	0,06	0,03	0,02	0,04	0,18	0,13	0,08	0,06	0,11	0,04	0,02	139,67
54	0,00	0,06	0,21	0,22	0,04	0,02	0,02	0,01	0,06	0,04	0,03	0,02	0,05	0,04	0,03	0,02	0,06	0,04	0,04	0,05	0,24	0,13	0,11	0,06	0,12	0,04	0,02	132,75
55	0,00	0,10	0,31	0,29	0,06	0,03	0,02	0,02	0,05	0,03	0,02	0,02	0,05	0,04	0,03	0,02	0,06	0,05	0,06	0,09	0,30	0,19	0,10	0,05	0,11	0,04	0,02	126,17
56	0,00	0,19	0,44	0,42	0,08	0,04	0,03	0,02	0,06	0,04	0,02	0,02	0,04	0,03	0,02	0,02	0,09	0,08	0,09	0,12	0,38	0,16	0,08	0,06	0,10	0,04	0,02	119,91
57	0,00	0,27	0,59	0,57	0,12	0,05	0,03	0,02	0,06	0,05	0,02	0,02	0,04	0,03	0,02	0,02	0,12	0,13	0,14	0,17	0,50	0,18	0,09	0,06	0,11	0,04	0,02	113,97
58	0,00	0,38	0,77	0,73	0,15	0,04	0,03	0,02	0,06	0,03	0,02	0,01	0,03	0,02	0,02	0,02	0,26	0,27	0,24	0,22	0,55	0,20	0,10	0,06	0,09	0,03	0,01	108,32
59	0,00	0,61	0,95	0,85	0,14	0,04	0,02	0,02	0,06	0,03	0,02	0,01	0,03	0,03	0,03	0,05	0,72	0,49	0,33	0,26	0,63	0,23	0,12	0,06	0,10	0,03	0,02	102,95
60	0,01	0,76	1,01	0,95	0,11	0,02	0,01	0,01	0,07	0,06	0,04	0,03	0,15	0,15	0,13	0,18	1,23	0,53	0,34	0,26	0,61	0,24	0,11	0,06	0,09	0,02	0,01	97,85
61	0,01	0,79	1,04	1,36	0,14	0,02	0,01	0,01	0,36	0,29	0,15	0,12	0,49	0,35	0,25	0,29	1,34	0,52	0,32	0,26	0,58	0,21	0,09	0,05	0,07	0,02	0,01	93,00
62	0,00	0,64	0,98	1,23	0,13	0,02	0,02	0,07	1,32	0,70	0,23	0,20	0,62	0,39	0,26	0,29	1,32	0,51	0,30	0,25	0,58	0,20	0,08	0,05	0,07	0,02	0,01	88,39
63	0,00	0,55	0,91	1,54	0,17	0,04	0,04	0,15	2,18	0,67	0,26	0,20	0,65	0,42	0,28	0,30	1,40	0,54	0,32	0,27	0,59	0,22	0,09	0,05	0,07	0,02	0,01	84,00
64	0,02	0,45	0,71	1,54	0,17	0,03	0,03	0,17	2,17	0,75	0,28	0,21	0,68	0,43	0,29	0,32	1,41	0,55	0,35	0,29	0,61	0,23	0,10	0,06	0,07	0,02	0,01	79,84
65	0,00	0,33	0,58	1,06	0,12	0,03	0,03	0,16	2,06	0,81	0,27	0,21	0,66	0,42	0,28	0,27	1,38	0,54	0,35	0,28	0,61	0,23	0,12	0,06	0,07	0,02	0,01	75,88
66	0,00	0,24	0,44	0,65	0,07	0,02	0,02	0,07	1,66	0,67	0,25	0,19	0,66	0,42	0,28	0,30	1,41	0,54	0,33	0,26	0,61	0,22	0,09	0,04	0,05	0,02	0,01	72,12
67	0,00	0,19	0,32	0,41	0,05	0,01	0,01	0,01	0,54	0,46	0,21	0,18	0,63	0,41	0,27	0,31	1,42	0,53	0,33	0,27	0,58	0,20	0,08	0,05	0,05	0,01	0,01	68,55
68	0,00	0,15	0,25	0,50	0,04	0,01	0,01	0,01	0,16	0,20	0,10	0,08	0,35	0,29	0,21	0,25	1,40	0,51	0,32	0,26	0,53	0,17	0,07	0,03	0,04	0,01	0,01	65,15
69	0,00	0,08	0,15	0,20	0,02	0,01	0,01	0,01	0,03	0,03	0,02	0,02	0,07	0,08	0,08	0,14	1,14	0,49	0,33	0,25	0,53	0,18	0,07	0,03	0,03	0,01	0,01	61,92
70	0,01	0,03	0,06	0,07	0,02	0,01	0,01	0,01	0,02	0,01	0,01	0,01	0,01	0,01	0,02	0,03	0,28	0,33	0,25	0,21	0,46	0,15	0,06	0,04	0,03	0,01	0,00	58,85
71	0,00	0,01	0,02	0,03	0,01	0,01	0,01	0,00	0,01	0,01	0,01	0,00	0,01	0,01	0,01	0,00	0,04	0,05	0,07	0,10	0,34	0,14	0,06	0,03	0,03	0,01	0,01	55,93
72	0,00	0,02	0,01	0,01	0,01	0,00	0,01	0,00	0,01	0,01	0,00	0,00	0,00	0,00	0,00	0,00	0,02	0,02	0,02	0,03	0,16	0,10	0,04	0,03	0,02	0,01	0,01	53,16
73	0,02	0,00	0,01	0,01	0,00	0,00	0,00	0,00	0,00	0,00	0,00	0,00	0,00	0,00	0,00	0,00	0,01	0,01	0,01	0,01	0,09	0,06	0,03	0,02	0,01	0,01	0,01	50,52
74	0,00	0,00	0,00	0,00	0,00	0,00	0,00	0,00	0,00	0,00	0,00	0,00	0,00	0,00	0,00	0,00	0,00	0,00	0,00	0,01	0,05	0,04	0,02	0,01	0,01	0,00	0,00	48,02

0,00	0,02	0,03	0,04	0,10	0,15	0,20	0,40	0,60	0,80	1,00	2,0	3,0	mg/ml
0,02	0,03	0,04	0,10	0,15	0,20	0,40	0,60	0,80	1,00	2,00	3,0	10,0	

Fractionation conditions and details of the Figure cf. Fig. 4c of the manuscript.

Protein concentrations derived from A_{280} are given by figures (mg/mL) and color coded.

Frames indicate 2D sub-fractions searched for PTM by the Proteome Discoverer©.

Fig. 4: Protein distribution of human CSF after 2D-AEC, section of albumin containing sub-fractions

2D→ 1D↓	0	1	2	3	4	5	6	7	8	9	10	11	12	13	14	15	16	17	18	19	20	21	22	Mr (kDa)
		sample			25 mM NaCl				40 mM NaCl				50 mM NaCl				75 mM NaCl				100 mM NaCl			
9	0.003	0.003	0.003	0.004	0.003	0.002	0.002	0.003	0.003	0.001	0.002	0.003	0.001	0.000	0.000	0.003	0.003	0.002	0.001	0.003	0.012	0.010	0.008	651.00
10	0.002	0.003	0.002	0.004	0.004	0.003	0.002	0.003	0.004	0.000	0.001	0.004	0.004	0.001	0.001	0.003	0.005	0.002	0.002	0.005	0.013	0.012	0.010	559.43
11	0.000	0.003	0.001	0.002	0.003	0.001	0.001	0.003	0.003	0.007	0.000	0.002	0.005	0.000	0.000	0.001	0.006	0.002	0.001	0.004	0.020	0.015	0.009	480.74
12	0.002	0.003	0.002	0.004	0.004	0.002	0.002	0.002	0.004	0.000	0.000	0.003	0.004	0.001	0.001	0.002	0.009	0.004	0.002	0.005	0.027	0.017	0.011	413.12
13	0.003	0.003	0.005	0.007	0.005	0.004	0.002	0.005	0.006	0.003	0.001	0.004	0.006	0.003	0.001	0.008	0.009	0.010	0.005	0.011	0.039	0.023	0.011	355.01
14	0.000	0.004	0.007	0.007	0.005	0.000	0.000	0.005	0.010	0.002	0.001	0.002	0.004	0.000	-0.001	0.004	0.023	0.013	0.011	0.021	0.044	0.021	0.015	305.07
15	0.001	0.023	0.035	0.028	0.010	0.004	0.002	0.008	0.017	0.008	0.003	0.003	0.009	0.004	0.001	0.008	0.024	0.017	0.011	0.020	0.042	0.020	0.011	262.16
16	0.002	0.112	0.177	0.113	0.038	0.020	0.015	0.017	0.036	0.017	0.011	0.010	0.018	0.011	0.007	0.024	0.034	0.022	0.018	0.022	0.049	0.025	0.016	225.29
17	0.012	0.278	0.378	0.227	0.053	0.028	0.020	0.022	0.043	0.021	0.014	0.014	0.021	0.014	0.010	0.013	0.037	0.022	0.017	0.022	0.043	0.025	0.018	193.60
18	0.004	0.197	0.345	0.229	0.036	0.023	0.016	0.017	0.035	0.017	0.011	0.010	0.017	0.011	0.006	0.009	0.029	0.017	0.010	0.016	0.031	0.019	0.013	166.37
19	0.003	0.100	0.187	0.127	0.027	0.016	0.014	0.015	0.029	0.017	0.011	0.012	0.016	0.010	0.008	0.012	0.026	0.016	0.008	0.012	0.033	0.017	0.010	142.97
20	0.010	0.040	0.088	0.078	0.031	0.025	0.022	0.043	0.078	0.024	0.014	0.014	0.024	0.018	0.013	0.017	0.031	0.016	0.014	0.023	0.055	0.030	0.016	122.86
21	0.002	0.041	0.155	0.155	0.097	0.080	0.069	0.080	0.168	0.037	0.018	0.016	0.032	0.020	0.015	0.018	0.186	0.184	0.135	0.121	0.208	0.082	0.033	105.57
22	0.002	0.092	0.333	0.336	0.164	0.102	0.071	0.068	0.507	0.480	0.208	0.164	0.458	0.320	0.208	0.241	0.676	0.219	0.139	0.136	0.291	0.128	0.047	90.72
23	0.003	0.096	0.307	0.360	0.132	0.076	0.078	0.162	1.622	0.769	0.225	0.154	0.446	0.332	0.193	0.195	0.693	0.240	0.155	0.134	0.322	0.146	0.059	77.96
24	0.003	0.081	0.238	0.277	0.087	0.043	0.031	0.051	1.260	0.747	0.238	0.157	0.454	0.311	0.201	0.201	0.702	0.232	0.152	0.131	0.321	0.139	0.060	67.00
25	0.005	0.064	0.148	0.119	0.048	0.031	0.027	0.045	0.372	0.307	0.167	0.164	0.427	0.267	0.172	0.252	0.594	0.173	0.107	0.116	0.217	0.086	0.054	57.57
26	0.002	0.029	0.060	0.049	0.020	0.013	0.009	0.011	0.019	0.008	0.005	0.006	0.011	0.009	0.009	0.022	0.403	0.228	0.124	0.090	0.144	0.062	0.024	49.47
27	0.003	0.098	0.138	0.085	0.018	0.006	0.004	0.004	0.012	0.003	0.002	0.003	0.005	0.002	0.001	0.003	0.015	0.028	0.044	0.060	0.078	0.024	0.009	42.52
28	0.014	0.251	0.316	0.193	0.040	0.009	0.005	0.005	0.010	0.006	0.003	0.004	0.005	0.005	0.002	0.003	0.009	0.010	0.012	0.018	0.037	0.016	0.007	36.54
29	0.012	0.255	0.314	0.201	0.015	0.007	0.006	0.007	0.009	0.004	0.003	0.006	0.005	0.003	0.002	0.005	0.007	0.008	0.007	0.011	0.020	0.009	0.005	31.40
30	0.005	0.132	0.159	0.100	0.021	0.006	0.002	0.004	0.007	0.003	0.002	0.002	0.002	0.000	-0.001	0.001	0.003	0.002	0.007	0.008	0.018	0.006	0.002	26.98
31	0.002	0.039	0.057	0.035	0.010	0.002	0.002	0.004	0.003	-0.001	0.001	0.002	0.001	0.000	0.001	0.002	0.002	0.000	0.002	0.003	0.008	0.001	0.003	23.19
32	0.004	0.009	0.024	0.021	0.009	0.004	0.002	0.004	0.006	0.002	0.000	0.003	0.002	0.000	-0.003	0.002	0.004	0.001	0.002	0.006	0.010	0.002	0.001	19.92

	0.00	0.02	0.03	0.04	0.10	0.15	0.20	0.40	0.60	0.80	1.00	2.00	3.00	mg/ml
	0.02	0.03	0.04	0.10	0.15	0.20	0.40	0.60	0.80	1.00	2.00	3.00	10.00	

Fractionation conditions and details of the Figure cf. Fig. 4c of the manuscript and Supplementary Fig. 3.
Frames indicate 2D sub-fractions analyzed for PTM by the Proteome Discoverer©.

Fig. 5: Concentrations of albumin determined by ELISA in human plasma (pools of sub-fractions) and human CSF sub-fractions

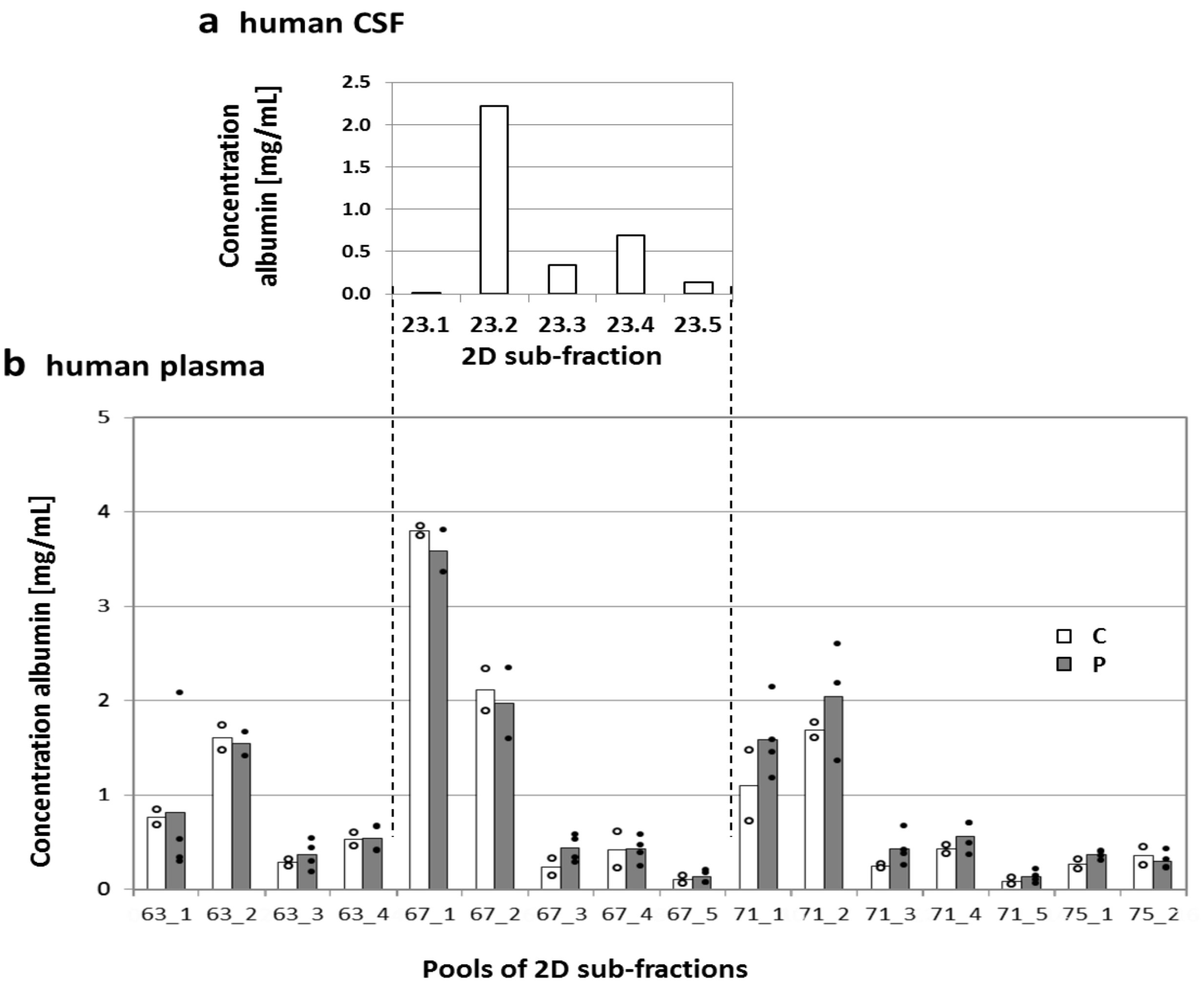

Nomenclature of sub-fractions and pools: see Supplementary Figs. 2a, 2b, and 3.

a: 2D sub-fractions from human CSF (concentrated mixture of 7 individuals without any neurological disease), mean values of two determinations.

b: Pools of sub-fractions from human plasma from healthy controls (C) and patients suffering from ESRD (P). Mean values of 2 individuals (C) and 4 individuals (P), determined each in duplicates and two different dilutions. Individual mean data points are given additionally.

Fig. 6: Comparison of specific AGE fluorescence intensities with MS quantifiers and AEC-elution

Pools with sub-fractions from human plasma were analyzed by MS and fluorescence measurement (cf. Online Methods). Data on human plasma were combined from individually pre-fractionated, prepared and analyzed samples from two healthy male volunteers (multi reports from four runs) and from four patients under hemodialysis (multi reports from eight runs). #: number of modification sites.

Trendlines and R^2 are given in the same color as and near to the corresponding data points., if applicable

■ □ , sub-fractions derived from centric albumin (1D 67); ▲ △ , sub-fractions derived from apparently LMW albumin (1D 71).

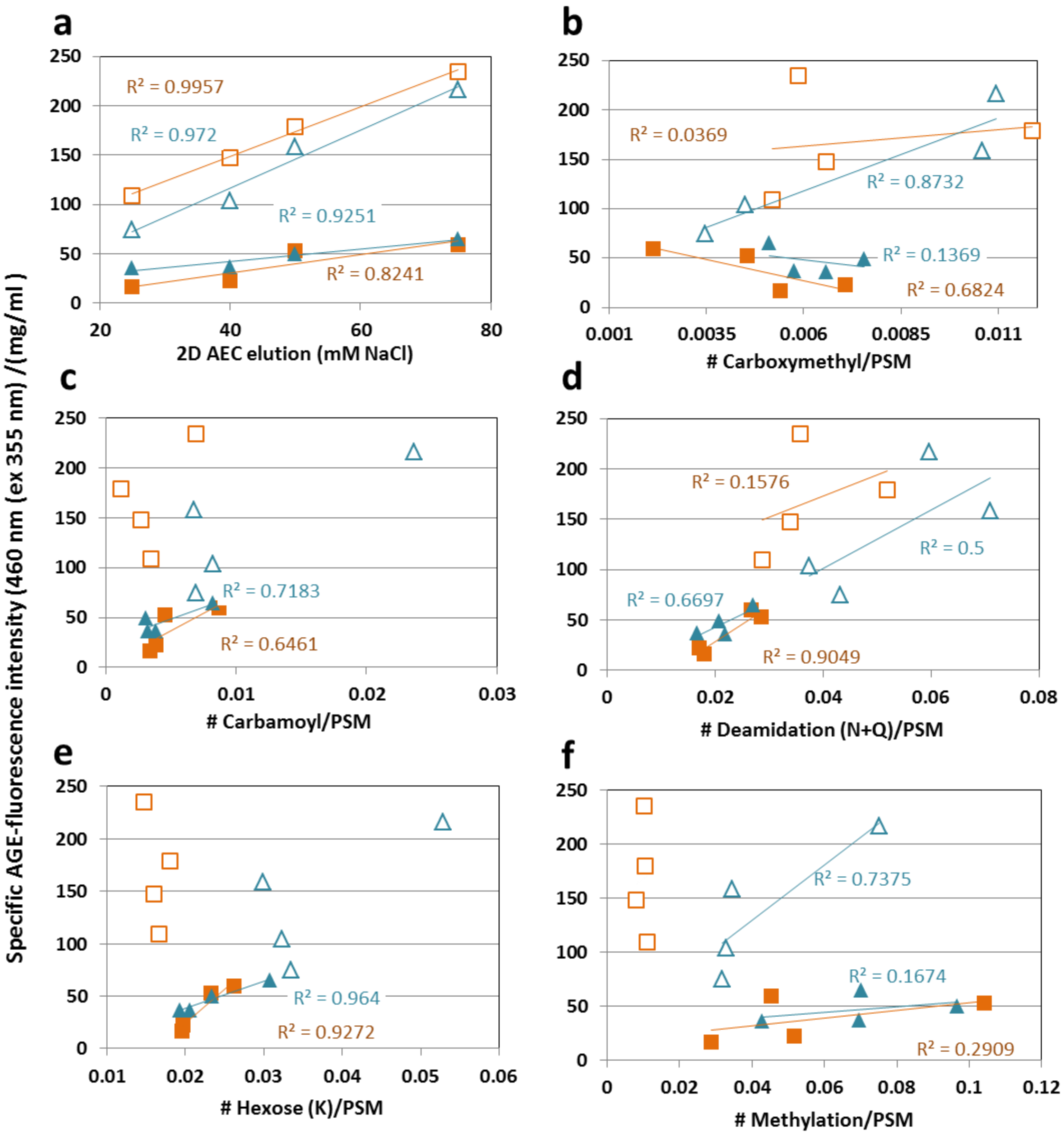

Fig. 7: Comparison of some MS quantifiers with immunolologic quantifiers for total albumin, glycated, and carbamylated albumin

Pools of sub-fractions from plasma were analyzed by MS and available ELISA-kits (cf. Online Methods)
Data of human plasma were combined from individually pre-fractionated, prepared and analyzed samples from two healthy male volunteers (multi reports from four runs) and from four patients under hemodialysis (multi reports from eight runs).
Trend lines and R^2 are given in the same color as and near to the corresponding data points.

a, b: all pools of sub-fractions analyzed from ◆ healthy individuals and ◇ patients with ESRD; Ordinates: mass spectrometric quantifier; modified sites per PSM (n PTM/PSM); Abscissa: albumin concentrations determined by the in-house ELISA.

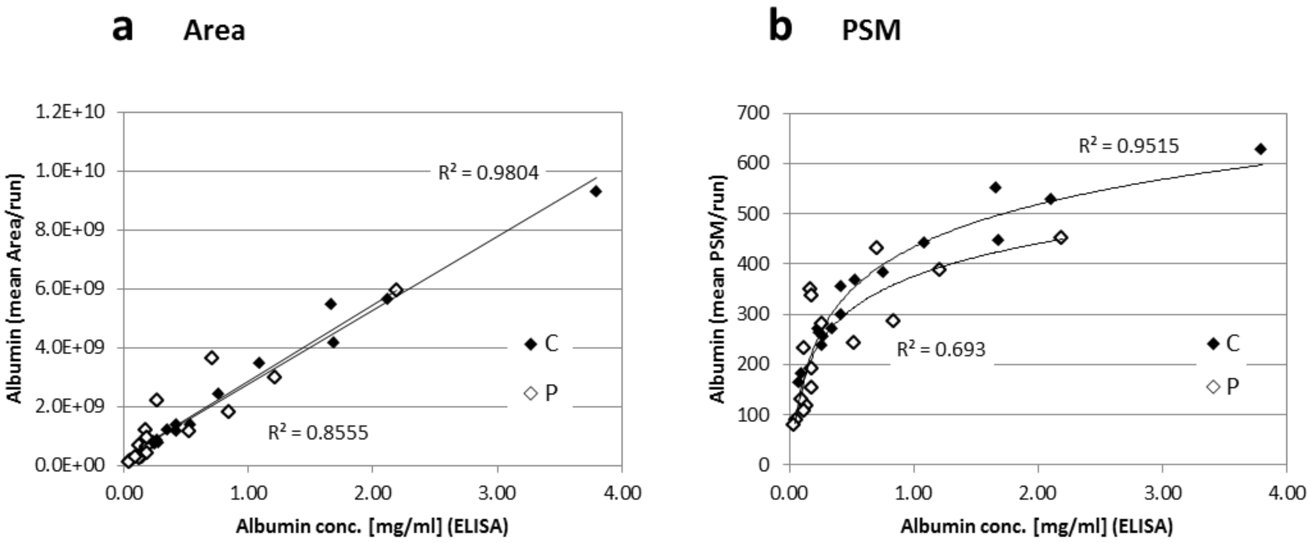

Fig. 8a: Protein distribution of human female plasma after 2D-AEC, section of albumin containing 2D sub-fractions

2D→	0	1	2	3	4	5	6	7	8	9	10	11	12	13	14	15	16	17	18	19	20	21	22	Mr
1D↓		sample			25 mM NaCl				40 mM NaCl				50 mM NaCl				75 mM NaCl				100 mM NaCl			[kDa]
41	0.01	0.20	0.32	0.31	0.08	0.03	0.02	0.01	0.04	0.03	0.02	0.02	0.04	0.03	0.03	0.03	0.14	0.11	0.09	0.10	0.28	0.15	0.08	300.93
42	0.02	0.27	0.41	0.40	0.09	0.03	0.03	0.01	0.05	0.03	0.02	0.02	0.04	0.03	0.02	0.03	0.13	0.10	0.08	0.08	0.24	0.12	0.07	285.42
43	0.03	0.45	0.63	0.59	0.16	0.04	0.03	0.02	0.06	0.03	0.02	0.02	0.04	0.03	0.02	0.03	0.11	0.09	0.07	0.08	0.20	0.11	0.07	270.71
44	0.04	0.71	0.96	0.93	0.19	0.05	0.04	0.02	0.07	0.03	0.02	0.02	0.04	0.03	0.02	0.03	0.11	0.08	0.06	0.07	0.20	0.10	0.06	256.76
45	0.08	1.01	1.33	1.26	0.26	0.07	0.05	0.03	0.09	0.04	0.03	0.02	0.05	0.03	0.03	0.03	0.11	0.08	0.06	0.07	0.18	0.10	0.07	243.53
46	0.08	1.38	1.74	1.68	0.35	0.08	0.05	0.03	0.09	0.05	0.03	0.03	0.05	0.04	0.03	0.03	0.11	0.08	0.06	0.06	0.19	0.11	0.06	230.98
47	0.20	1.73	1.97	1.84	0.31	0.07	0.06	0.04	0.10	0.04	0.03	0.03	0.06	0.04	0.03	0.04	0.12	0.07	0.06	0.07	0.20	0.11	0.07	219.07
48	0.28	1.77	2.01	1.87	0.46	0.08	0.05	0.03	0.07	0.04	0.03	0.03	0.05	0.04	0.03	0.04	0.13	0.09	0.07	0.07	0.24	0.13	0.08	207.78
49	0.14	1.80	2.00	1.93	0.39	0.07	0.05	0.03	0.09	0.04	0.03	0.02	0.05	0.04	0.03	0.03	0.11	0.08	0.06	0.07	0.23	0.13	0.07	197.07
50	0.13	1.69	1.94	1.87	0.37	0.07	0.05	0.03	0.09	0.04	0.03	0.02	0.05	0.04	0.03	0.03	0.11	0.08	0.06	0.07	0.23	0.13	0.07	186.92
51	0.09	1.52	1.77	1.69	0.26	0.06	0.04	0.02	0.08	0.04	0.02	0.02	0.04	0.03	0.03	0.03	0.10	0.07	0.06	0.07	0.22	0.12	0.06	177.28
52	0.13	1.16	1.39	1.52	0.25	0.05	-0.01	0.02	0.07	0.04	0.03	0.02	0.11	0.05	0.04	0.04	0.11	0.07	0.06	0.08	0.21	0.12	0.07	168.15
53	0.06	0.84	1.07	1.02	0.16	0.03	0.03	0.02	0.07	0.03	0.02	0.02	0.05	0.03	0.02	0.02	0.09	0.06	0.05	0.06	0.21	0.10	0.05	159.48
54	0.03	0.57	0.72	0.68	0.12	0.04	0.03	0.02	0.08	0.04	0.03	0.02	0.06	0.04	0.03	0.03	0.09	0.06	0.05	0.06	0.20	0.10	0.05	151.26
55	0.02	0.35	0.47	0.46	0.09	0.04	0.03	0.02	0.09	0.05	0.03	0.02	0.07	0.04	0.03	0.02	0.09	0.06	0.05	0.06	0.18	0.09	0.04	143.47
56	0.02	0.23	0.34	0.36	0.10	0.04	0.04	0.01	0.06	0.03	0.02	0.03	0.08	0.05	0.03	0.03	0.12	0.07	0.06	0.06	0.19	0.09	0.05	136.07
57	0.01	0.15	0.31	0.34	0.11	0.06	0.06	0.01	0.09	0.04	0.02	0.03	0.10	0.06	0.04	0.03	0.16	0.08	0.06	0.06	0.18	0.08	0.04	129.06
58	0.01	0.16	0.36	0.39	0.14	0.10	0.09	0.08	0.22	0.06	0.03	0.04	0.10	0.05	0.04	0.06	0.21	0.09	0.06	0.08	0.18	0.06	0.03	122.41
59	0.00	0.11	0.39	0.50	0.22	0.15	0.13	0.14	0.39	0.11	0.05	0.04	0.11	0.07	0.06	0.08	0.28	0.13	0.08	0.08	0.21	0.08	0.04	116.10
60	0.00	0.11	0.45	0.60	0.30	0.19	0.16	0.18	0.39	0.13	0.07	0.04	0.13	0.13	0.11	0.12	0.33	0.14	0.08	0.09	0.21	0.07	0.04	110.12
61	0.00	0.11	0.48	0.69	0.36	0.23	0.18	0.18	0.49	0.29	0.16	0.08	0.29	0.22	0.14	0.14	0.39	0.18	0.10	0.10	0.23	0.09	0.04	104.44
62	0.00	0.11	0.56	0.81	0.40	0.22	0.15	0.15	0.95	0.47	0.20	0.17	0.36	0.24	0.14	0.14	0.42	0.19	0.11	0.11	0.23	0.09	0.04	99.06
63	0.00	0.14	0.61	0.89	0.45	0.21	0.21	0.27	1.37	0.52	0.18	0.15	0.36	0.25	0.15	0.14	0.44	0.21	0.12	0.10	0.23	0.10	0.04	93.95
64	0.00	0.12	0.61	0.94	0.47	0.18	0.21	0.59	1.57	0.52	0.17	0.15	0.37	0.25	0.15	0.15	0.47	0.22	0.12	0.11	0.24	0.10	0.04	89.11
65	0.00	0.22	0.77	1.49	0.59	0.19	0.22	0.70	1.61	0.52	0.18	0.15	0.38	0.25	0.14	0.14	0.49	0.22	0.12	0.10	0.25	0.10	0.04	84.52
66	0.00	0.11	0.60	1.71	1.01	0.43	0.45	0.91	1.69	0.52	0.17	0.16	0.38	0.24	0.16	0.16	0.51	0.22	0.12	0.12	0.26	0.11	0.05	80.16
67	0.00	0.16	0.87	1.92	1.01	0.48	0.46	0.68	1.63	0.54	0.20	0.17	0.39	0.24	0.15	0.15	0.51	0.23	0.12	0.11	0.27	0.11	0.04	76.03
68	0.00	0.05	0.60	2.01	1.19	0.48	0.48	0.96	1.68	0.51	0.16	0.15	0.36	0.25	0.14	0.15	0.52	0.22	0.12	0.11	0.28	0.11	0.04	72.11
69	0.00	0.05	0.54	1.97	1.10	0.46	0.48	0.85	1.63	0.48	0.17	0.15	0.39	0.23	0.14	0.14	0.51	0.21	0.12	0.10	0.29	0.12	0.04	68.40
70	0.00	0.04	0.42	1.87	1.10	0.45	0.47	0.84	1.64	0.52	0.18	0.15	0.39	0.25	0.15	0.15	0.53	0.22	0.12	0.10	0.29	0.12	0.05	64.87
71	0.00	0.03	0.26	1.32	0.91	0.38	0.42	0.73	1.70	0.50	0.16	0.15	0.37	0.23	0.14	0.15	0.49	0.20	0.10	0.09	0.25	0.10	0.04	61.53
72	0.00	0.03	0.24	0.78	0.55	0.29	0.33	0.79	1.66	0.56	0.15	0.15	0.35	0.23	0.14	0.14	0.43	0.18	0.10	0.08	0.21	0.09	0.03	58.36
73	0.00	0.03	0.12	0.41	0.37	0.25	0.30	0.73	1.54	0.51	0.14	0.15	0.37	0.21	0.13	0.15	0.40	0.16	0.09	0.08	0.19	0.08	0.03	55.35
74	0.00	0.01	0.07	0.14	0.12	0.09	0.12	0.37	1.68	0.52	0.17	0.15	0.38	0.24	0.14	0.15	0.44	0.18	0.09	0.08	0.18	0.07	0.03	52.50
75	0.00	0.00	0.04	0.08	0.06	0.04	0.06	0.79	1.60	0.53	0.18	0.16	0.37	0.24	0.15	0.14	0.42	0.17	0.09	0.07	0.16	0.07	0.02	49.79
76	0.00	0.00	0.02	0.03	0.02	0.01	0.01	0.72	1.49	0.51	0.18	0.16	0.37	0.23	0.15	0.16	0.41	0.15	0.08	0.06	0.12	0.05	0.02	47.22
77	0.00	0.00	0.01	0.01	0.01	0.01	0.01	0.03	0.63	0.43	0.18	0.14	0.37	0.24	0.13	0.15	0.34	0.13	0.06	0.04	0.09	0.03	0.01	44.79
78	0.00	0.00	0.00	0.00	0.01	0.01	0.00	0.00	0.01	0.03	0.03	0.01	0.05	0.09	0.09	0.12	0.32	0.11	0.05	0.04	0.06	0.02	0.01	42.48

0.00	0.02	0.03	0.04	0.10	0.15	0.2	0.4	0.6	0.8	1.0	2.0	3.0	mg/ml
0.02	0.03	0.04	0.10	0.15	0.20	0.4	0.6	0.8	1.0	2.0	3.0	10.0	

Protein concentrations derived from A_{280} are given by figures (mg/mL) and color coded, cf. Fig. 4c of the manuscript and Supplementary Figure 8b.

Frames indicate pools of 2D sub-fractions analyzed for PTM by the Proteome Discoverer©.

Fig. 8b: PTM of albumin (P02768) in the pools of 2D-sub-fractions from human plasma from two female healthy volunteers.

The numbers of 1D and 2D sub-fractions (cf. Supplementary Fig. S8a) are given in the left column and upper line of this figure. Pools were built from 3 to 8 sub-fractions and denominated in the first section of this Figure. Only three dynamic PTM were determined simultaneously by the Proteome Discoverer© multiconsensus analysis of four runs (two samples, analyzed by mass spec in duplicates each, cf. Methods). The type of PTM is given in the head of each section. Data given are the numbers (#) of **modified residues per PSM** (total number of identified peptide spectra matched for albumin). The data are further visualized by a grey code.

Nomenclature of pools containing 3 to 8 2D sub-fractions

2D→	2	3	8	12	16	20
1D↓						
62						
63			63.2		63.4	
64						
65						
66						67.5
67	67.1		67.2	67.3	67.4	
68						
69						
70						
71	71.1		71.2	71.3	71.4	
72						
73						
74			75.2			
75				75.3	75.4	
76						
77						
78						

# Palmitoyl

2D→	2	3	8	12	16	20
1D↓						
62						
63			0.0023		0.0016	
64						
65						
66						0.0016
67	0.0048		0.0050	0.0056	0.0013	
68						
69						
70						
71	0.0028		0.0026	0.0016	0.0013	
72						
73						
74			0.0010			
75				0.0017	0.0031	
76						
77						
78						

Sum [# methylation, # dimethylation, # ethylation, # propionylation, and # acetylation]

2D→	2	3	8	12	16	20
1D↓						
62						
63			0.0499		0.0292	
64						
65						
66						0.0814
67	0.0270		0.0280	0.0398	0.0309	
68						
69						
70						
71	0.0309		0.0389	0.0301	0.0283	
72						
73						
74			0.0367			
75				0.0632	0.0505	
76						
77						
78						

Sum [# octanoyl, # decanoyl, # myristoyl, # palmitoyl, # palmitoleyl]

2D→	2	3	8	12	16	20
1D↓						
62						
63			0.0052		0.0031	
64						
65						
66						0.0050
67	0.0082		0.0086	0.0091	0.0020	
68						
69						
70						
71	0.0054		0.0036	0.0025	0.0013	
72						
73						
74			0.0010			
75				0.0050	0.0045	
76						
77						
78						

# Myristoyl

2D→	2	3	8	12	16	20
1D↓						
62						
63			0.0012		0.0016	
64						
65						
66						0.0016
67	0.0018		0.0032	0.0021	0.0007	
68						
69						
70						
71	0.0008		0.0010	0.0008	0.0000	
72						
73						
74			0.0000			
75				0.0017	0.0007	
76						
77						
78						

Sum # deamidations of N and Q

2D→	2	3	8	12	16	20
1D↓						
62						
63			0.024		0.027	
64						
65						
66						0.050
67	0.016		0.020	0.029	0.025	
68						
69						
70						
71	0.017		0.026	0.050	0.027	
72						
73						
74			0.026			
75				0.021	0.038	
76						
77						
78						

# Deamidation of R

<table>
<tr><th>2D→</th><th>2</th><th>3</th><th>8</th><th>12</th><th>16</th><th>20</th></tr>
<tr><td>1D↓</td><td></td><td></td><td></td><td></td><td></td><td></td></tr>
<tr><td>62</td><td></td><td></td><td rowspan="4">0.011</td><td></td><td rowspan="4">0.012</td><td></td></tr>
<tr><td>63</td><td></td><td></td><td></td><td></td></tr>
<tr><td>64</td><td></td><td></td><td></td><td rowspan="6">0.015</td></tr>
<tr><td>65</td><td></td><td></td><td></td></tr>
<tr><td>66</td><td colspan="2" rowspan="4">0.010</td><td rowspan="4">0.009</td><td rowspan="4">0.010</td><td rowspan="4">0.009</td></tr>
<tr><td>67</td></tr>
<tr><td>68</td></tr>
<tr><td>69</td></tr>
<tr><td>70</td><td colspan="2" rowspan="3">0.007</td><td rowspan="3">0.011</td><td rowspan="4">0.024</td><td rowspan="4">0.013</td><td></td></tr>
<tr><td>71</td><td></td></tr>
<tr><td>72</td><td></td></tr>
<tr><td>73</td><td></td><td></td><td rowspan="4">0.012</td><td></td></tr>
<tr><td>74</td><td></td><td></td><td rowspan="4">0.008</td><td rowspan="4">0.011</td><td></td></tr>
<tr><td>75</td><td></td><td></td><td></td></tr>
<tr><td>76</td><td></td><td></td><td></td></tr>
<tr><td>77</td><td></td><td></td><td></td><td></td></tr>
<tr><td>78</td><td></td><td></td><td></td><td></td><td></td><td></td></tr>
</table>

# Hexose on K

<table>
<tr><th>2D→</th><th>2</th><th>3</th><th>8</th><th>12</th><th>16</th><th>20</th></tr>
<tr><td>1D↓</td><td></td><td></td><td></td><td></td><td></td><td></td></tr>
<tr><td>62</td><td></td><td></td><td rowspan="4">0.0182</td><td></td><td rowspan="4">0.0231</td><td></td></tr>
<tr><td>63</td><td></td><td></td><td></td><td></td></tr>
<tr><td>64</td><td></td><td></td><td></td><td rowspan="6">0.0126</td></tr>
<tr><td>65</td><td></td><td></td><td></td></tr>
<tr><td>66</td><td colspan="2" rowspan="4">0.0137</td><td rowspan="4">0.0185</td><td rowspan="4">0.0230</td><td rowspan="4">0.0214</td></tr>
<tr><td>67</td></tr>
<tr><td>68</td></tr>
<tr><td>69</td></tr>
<tr><td>70</td><td colspan="2" rowspan="3">0.0132</td><td rowspan="3">0.0176</td><td rowspan="4">0.0175</td><td rowspan="4">0.0212</td><td></td></tr>
<tr><td>71</td><td></td></tr>
<tr><td>72</td><td></td></tr>
<tr><td>73</td><td></td><td></td><td rowspan="4">0.0205</td><td></td></tr>
<tr><td>74</td><td></td><td></td><td rowspan="4">0.0175</td><td rowspan="4">0.0237</td><td></td></tr>
<tr><td>75</td><td></td><td></td><td></td></tr>
<tr><td>76</td><td></td><td></td><td></td></tr>
<tr><td>77</td><td></td><td></td><td></td><td></td></tr>
<tr><td>78</td><td></td><td></td><td></td><td></td><td></td><td></td></tr>
</table>

Sum [# carboxymethyl and # carboxyethyl on K]

<table>
<tr><th>2D→</th><th>2</th><th>3</th><th>8</th><th>12</th><th>16</th><th>20</th></tr>
<tr><td>1D↓</td><td></td><td></td><td></td><td></td><td></td><td></td></tr>
<tr><td>62</td><td></td><td></td><td rowspan="4">0.0076</td><td></td><td rowspan="4">0.0116</td><td></td></tr>
<tr><td>63</td><td></td><td></td><td></td><td></td></tr>
<tr><td>64</td><td></td><td></td><td></td><td rowspan="6">0.0089</td></tr>
<tr><td>65</td><td></td><td></td><td></td></tr>
<tr><td>66</td><td colspan="2" rowspan="4">0.0055</td><td rowspan="4">0.0051</td><td rowspan="4">0.0061</td><td rowspan="4">0.0064</td></tr>
<tr><td>67</td></tr>
<tr><td>68</td></tr>
<tr><td>69</td></tr>
<tr><td>70</td><td colspan="2" rowspan="3">0.0097</td><td rowspan="3">0.0085</td><td rowspan="4">0.0117</td><td rowspan="4">0.0076</td><td></td></tr>
<tr><td>71</td><td></td></tr>
<tr><td>72</td><td></td></tr>
<tr><td>73</td><td></td><td></td><td rowspan="4">0.0102</td><td></td></tr>
<tr><td>74</td><td></td><td></td><td rowspan="4">0.0091</td><td rowspan="4">0.0102</td><td></td></tr>
<tr><td>75</td><td></td><td></td><td></td></tr>
<tr><td>76</td><td></td><td></td><td></td></tr>
<tr><td>77</td><td></td><td></td><td></td><td></td></tr>
<tr><td>78</td><td></td><td></td><td></td><td></td><td></td><td></td></tr>
</table>

# Carbamoyl on K

<table>
<tr><th>2D→</th><th>2</th><th>3</th><th>8</th><th>12</th><th>16</th><th>20</th></tr>
<tr><td>1D↓</td><td></td><td></td><td></td><td></td><td></td><td></td></tr>
<tr><td>62</td><td></td><td></td><td rowspan="4">0.0022</td><td></td><td rowspan="4">0.0060</td><td></td></tr>
<tr><td>63</td><td></td><td></td><td></td><td></td></tr>
<tr><td>64</td><td></td><td></td><td></td><td rowspan="6">0.0000</td></tr>
<tr><td>65</td><td></td><td></td><td></td></tr>
<tr><td>66</td><td colspan="2" rowspan="4">0.0004</td><td rowspan="4">0.0034</td><td rowspan="4">0.0065</td><td rowspan="4">0.0066</td></tr>
<tr><td>67</td></tr>
<tr><td>68</td></tr>
<tr><td>69</td></tr>
<tr><td>70</td><td colspan="2" rowspan="3">0.0009</td><td rowspan="3">0.0018</td><td rowspan="4">0.0028</td><td rowspan="4">0.0034</td><td></td></tr>
<tr><td>71</td><td></td></tr>
<tr><td>72</td><td></td></tr>
<tr><td>73</td><td></td><td></td><td rowspan="4">0.0013</td><td></td></tr>
<tr><td>74</td><td></td><td></td><td rowspan="4">0.0031</td><td rowspan="4">0.0050</td><td></td></tr>
<tr><td>75</td><td></td><td></td><td></td></tr>
<tr><td>76</td><td></td><td></td><td></td></tr>
<tr><td>77</td><td></td><td></td><td></td><td></td></tr>
<tr><td>78</td><td></td><td></td><td></td><td></td><td></td><td></td></tr>
</table>

Fluorescence intensity at 460 nm (ex 355 nm)/(mg/ml) protein conc.

<table>
<tr><th>2D→</th><th>2</th><th>3</th><th>8</th><th>12</th><th>16</th><th>20</th></tr>
<tr><td>1D↓</td><td></td><td></td><td></td><td></td><td></td><td></td></tr>
<tr><td>62</td><td></td><td></td><td rowspan="4">n. d.</td><td></td><td rowspan="4">n. d.</td><td></td></tr>
<tr><td>63</td><td></td><td></td><td></td><td></td></tr>
<tr><td>64</td><td></td><td></td><td></td><td rowspan="6">0.00</td></tr>
<tr><td>65</td><td></td><td></td><td></td></tr>
<tr><td>66</td><td colspan="2" rowspan="4">5.78</td><td rowspan="4">6.81</td><td rowspan="4">126.77</td><td rowspan="4">32.58</td></tr>
<tr><td>67</td></tr>
<tr><td>68</td></tr>
<tr><td>69</td></tr>
<tr><td>70</td><td colspan="2" rowspan="3">n. d.</td><td rowspan="3">n. d.</td><td rowspan="4">n. d.</td><td rowspan="4">n. d.</td><td></td></tr>
<tr><td>71</td><td></td></tr>
<tr><td>72</td><td></td></tr>
<tr><td>73</td><td></td><td></td><td rowspan="4">n. d.</td><td></td></tr>
<tr><td>74</td><td></td><td></td><td rowspan="4">n. d.</td><td rowspan="4">n. d.</td><td></td></tr>
<tr><td>75</td><td></td><td></td><td></td></tr>
<tr><td>76</td><td></td><td></td><td></td></tr>
<tr><td>77</td><td></td><td></td><td></td><td></td></tr>
<tr><td>78</td><td></td><td></td><td></td><td></td><td></td><td></td></tr>
</table>

n. d.: not determined

Concentration parameters derived from A_{280} and mass spec counts

Total protein concentration (mg/ml, A_{280})

2D→ / 1D↓	2	3	8	12	16	20
62						
63			1.10		0.42	
64						
65						
66						0.28
67	1.93		1.32	0.39	0.51	
68						
69						
70						
71	1.43		1.39	0.38	0.40	
72						
73						
74			1.28			
75				0.31	0.36	
76						
77						
78						

PSM (albumin), mean and (SD)*

2D→ / 1D↓	2	3	8	12	16	20
62						
63			869.7 (69.9)		668.8 (50.2)	
64						
65						
66						269.4 (30.6)
67	1328.5 (68.8)		1079.6 (67.9)	691.8 (49.2)	752.6 (55.3)	
68						
69						
70						
71	1121.3 (74.8)		558.0 (49.9)	410.6 (23.3)	552.7 (53.4)	
72						
73						
74			623.7 (34.7)			
75				624.6 (54.5)	659.8 (53.6)	
76						
77						
78						

Ratio (%) of concentrations of albumin (ELISA)/total protein (A_{280})

2D→ / 1D↓	2	3	8	12	16	20
62						
63			63.10		76.79	
64						
65						
66						17.80
67	95.61		n. d.	n. d.	n. d.	
68						
69						
70						
71	78.03		n. d.	n. d.	n. d.	
72						
73						
74			76.55			
75				80.11	64.08	
76						
77						
78						

n. d.: not determined

Sequence coverage of albumin, mean and (SD) (%)*

2D→ / 1D↓	2	3	8	12	16	20
62						
63			82.23 (1.19)		80.78 (1.58)	
64						
65						
66						75.98 (2.18)
67	87.50 (0.89)		84.23 (0.90)	80.91 (1.08)	80.43 (1.57)	
68						
69						
70						
71	84.01 (0.97)		79.08 (1.52)	76.21 (1.82)	77.23 (1.33)	
72						
73						
74			77.49 (1.61)			
75				79.50 (1.97)	80.47 (1.70)	
76						
77						
78						

Protein of secondly highest abundance within the same sub-fraction (according coverage and MS signal intensities (area)

2D→ / 1D↓	2	3	8	12	16	20
62						
63			VDBP, Trf		A1AT, A2HS GP	
64						
65						
66						A2HSGP, A1AT, A1AGP, ApoAI, II
67	Trf		A1AT, VDBP	A1AT	A1AT	
68						
69						
70						
71	Trf, VDBP		Trfe, VDBP	A1AT, SP I	A1AT	
72						
73						
74			VDBP			
75				VDBP	A1AT	
76						
77						
78						

Abbreviations:

Trf, transferrin; Apo, apo-lipoprotein; A1AT, alpha-1-antitrypsin; A2HSGP, alpha-2-HS-glycoprotein; A1AGP, alpha-1-acid glycoprotein; VDBP, vitamin D-binding protein; SP I, serine protease I

*Mean and standard deviation of coverages and PSMs after 9 search runs included here.
